# cDC1 Subtype‐Specific In Vivo Targeting of Liposomes

**DOI:** 10.1002/advs.202515402

**Published:** 2026-01-28

**Authors:** Maximilian Schaaf, Michael Fichter, Lin Jian, Felicia Schön, Carina Jung, Paul Schneider, Kai Speth, Ana Mateos‐Maroto, Volker Mailänder, Kaloian Koynov, Svenja Morsbach, Katharina Landfester

**Affiliations:** ^1^ Max Planck Institute for Polymer Research Mainz Germany; ^2^ Department of Dermatology University Medical Center of the Johannes Gutenberg University Mainz Mainz Germany

**Keywords:** active targeting, CLEC9A, dendritic cells, in vivo, liposomes

## Abstract

Targeted modulation of immune cells holds great promise for improving therapeutic efficacy and minimizing systemic side effects; however, current strategies with nanocarriers often rely on untargeted or labor‐intensive approaches. To bridge this gap, combining clinically approved nanocarrier systems like liposomes with highly specific targeting ligands provides a promising approach that could reach clinical application faster than other carriers. However, while studies have shown the potential of ligand‐functionalized liposomes, most either lack sufficient immune cell subtype specificity or fail to translate their success from in vitro studies to the more complex environment in vivo. Therefore, a targeting approach was developed, based on a broad understanding of the colloidal system and functionalization procedure through an iterative process of physicochemical characterization and in vitro cell uptake studies. Liposomes were site‐specifically functionalized with varying ratios of anti‐CD11c or anti‐CLEC9A antibodies, targeting receptors on conventional type 1 dendritic cells (cDC1). After identification of favorable particle parameters in vitro, optimized constructs were tested in in vivo targeting experiments. Flow cytometry revealed significantly enhanced uptake of anti‐CD11c antibody‐functionalized liposomes into various dendritic cell subtypes, while anti‐CLEC9A antibody‐functionalized liposomes showed significantly enhanced uptake only in cDC1 cells. In conclusion, a liposome‐based cDC1 subtype‐specific nanocarrier was developed and applied in vivo.

## Graphical abstract

Dendritic cells, particularly the cDC1 subtype, offer a promising target for drug delivery via liposomes due to their pivotal role in immune regulation, allowing for amplified therapeutic responses. Herein, we show an integration of physicochemical characterization and cell experiments to achieve effective in vivo cDC1 targeting through anti‐CLEC9A antibody‐functionalized liposomes.

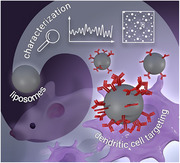

## 1 Introduction

In the field of nanocarriers (NCs) for drug delivery, a general distinction is made between passive and active targeting. While the result of passive targeting is mostly determined by carrier size, active targeting can employ energy‐dependent biological transport processes to overcome physical barriers. This can be achieved through attachment of a high‐affinity ligand, such as signaling molecules, carbohydrates, peptides, proteins, antibodies, aptamers, or oligonucleotides to the nanocarrier surface [1]. Ligand density has to be optimized to avoid scavenging by the mononuclear phagocyte system, while assuring targeting efficiency and optimal cell uptake [2, 3].

Because of their regulatory function within the immune system, dendritic cells (DCs) present a pivotal point in immunotherapy. For the active targeting of NCs in vivo, DCs offer a variety of cell surface receptors that can be addressed as targets. Among them, the classical DC‐marker CD11c can be found on various DC types, including conventional DCs (cDCs) [4]. Additionally, for DC subtype‐specific targeting, CLEC9A and its human ortholog DNGR1 have recently emerged as very promising targets [5]. Notably, their high sequence conservation indicates good translatability from murine models to clinical application [6]. CLEC9A is expressed almost exclusively by cDC1 cells, which can cross‐present antigens far more efficiently than other cell types and are therefore especially interesting for boosting immune responses [7]. To achieve targeting of DCs and especially cDC1 cells, liposomes are promising as they are a very well‐established carrier system already. They allow varying compositions and functionalization, provide easy and scalable manufacturing options, and possess a well‐known clinical safety profile [8]. Their involvement could mean reaching clinical application faster than with less established formulations.

However, despite the immense amount of research in this field, there is currently no actively targeted liposome construct with full FDA approval, though some are in ongoing clinical trials [9, 10]. This highlights the difficulty of the transfer from in vitro studies to successful in vivo application, which is partly due to increased detection by the immune system [9, 11]. And while there have been approaches to use anti‐CLEC9A antibody‐functionalized NCs, such as lipid nanoparticles or magnetite‐hydroxyethyl starch nanoparticles to target DC subsets in the past [12, 13], to the best of our knowledge, there are no reports of any comparable approaches for the case of liposomes as of today.

**FIGURE 1:**
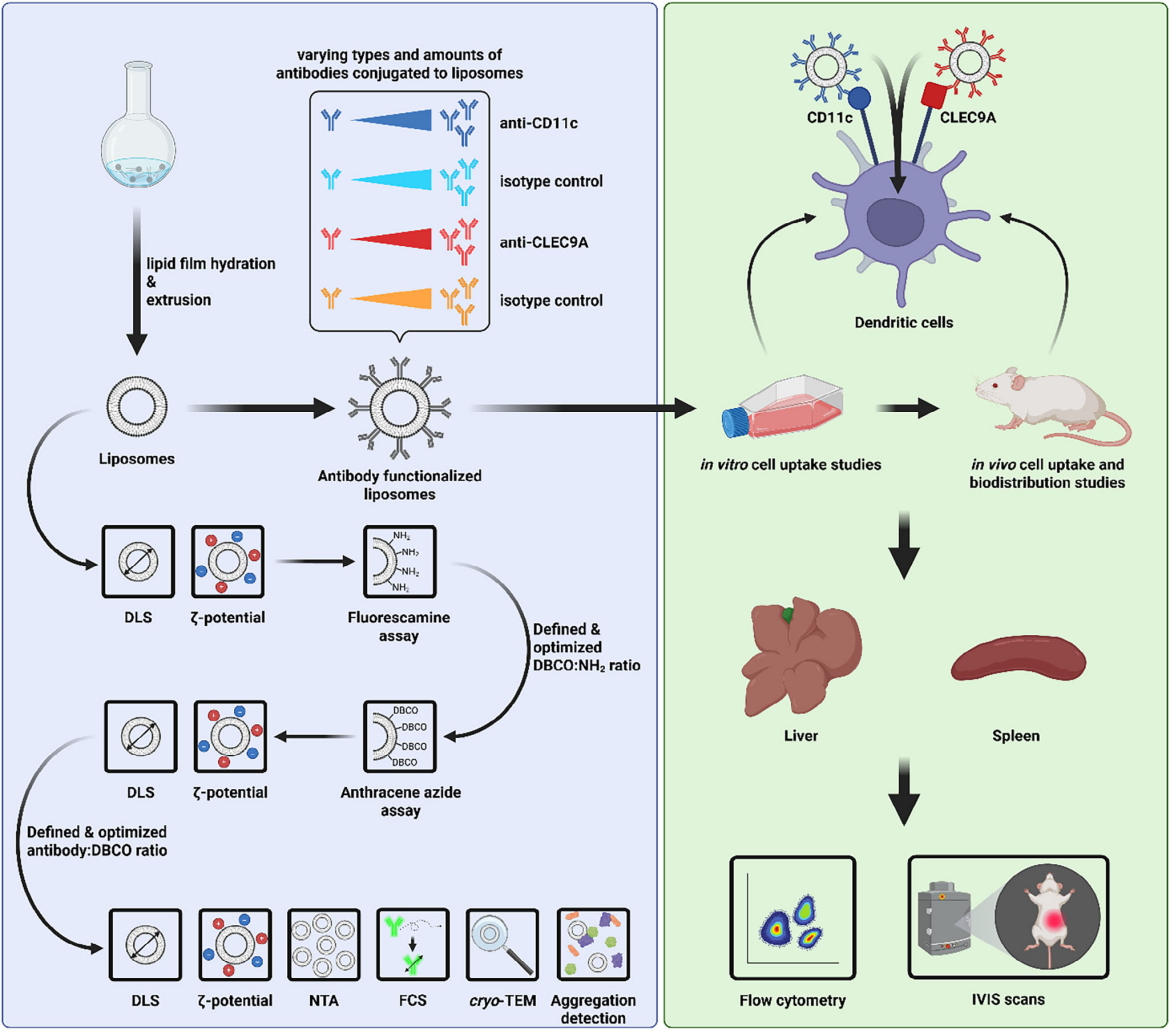
Schematic summary showing the complete workflow of the study. Liposome surface functionalization with varying types and amounts of antibodies was optimized through combined insights from broad physicochemical characterization and in vitro studies. Subsequently, optimized antibody‐liposome constructs were used for in vivo cell uptake and biodistribution studies, with analyses on the organ and cell level. DLS: dynamic light scattering, NTA: nanoparticle tracking analysis, FCS: fluorescence correlation spectroscopy, *cryo*‐TEM: *cryo* transmission electron microscopy. Created in BioRender. Schaaf, M. (2025) https://BioRender.com/vr1j9xi.

Therefore, we believe that DC targeting in general via CD11c and DC subtype‐specific targeting of liposomes via the CLEC9A surface receptor holds an enormous potential for highly specific and effective immunotherapeutic applications. In this study, we aimed to achieve in vivo cDC and cDC1 subtype‐specific targeting via anti‐CD11c and anti‐CLEC9A antibody‐functionalized liposomes, respectively, together with thorough characterization of relevant physicochemical particle parameters (see Figure 1). To this end, a two‐step surface functionalization strategy was employed and monitored through functional group quantification assays. After functionalization, the presence of specific antibody types was confirmed via fluorescence correlation spectroscopy (FCS), and relevant parameters for reaction control and colloidal stability were readily monitored and reported. Finally, in vitro studies were conducted to find the optimal formulation for successful in vivo application.

## 2 Results and Discussion

For the development and optimization of the cDC1 targeting strategy, liposomes composed of eggPC, DOPE, and cholesterol (ratio 1:1:1) were produced. This composition was shown to generally yield good cellular uptake in dendritic cells (DC2.4) without any functionalization [14]. Using this characteristic, the aim was to further enhance specificity by covalently attaching DC subtype‐specific antibodies on the liposomal surface, using a previously published procedure based on a strain‐promoted azide‐alkyne cycloaddition (SPAAC) [15].

### 2.1 Liposome Functionalization and Physicochemical Characterization

To achieve DC subtype‐specific targeting, we aimed at liposome functionalization with antibodies in an oriented manner. Based on a previously established functionalization strategy, antibodies were site‐specifically azidated to later be conjugated to strained alkyne groups on the liposomal surface in an oriented manner, ensuring full accessibility of their target recognition region [15]. The relevance of this approach was demonstrated in previous studies, which showed that site‐specific and oriented antibody functionalization strategies significantly enhance nanocarrier targeting when compared to unspecific and non‐directed functionalization approaches [16, 17]. Alternatively, Ju et al. recently demonstrated oriented functionalization with bispecific antibodies, with one antigen binding site anchoring the antibody to the carrier surface, enabling B‐cell targeting of liposomes without the need for covalent conjugation [18]. This however, requires a PEGylated carrier for anchoring, which may not always be desired or accessible.

**FIGURE 2:**
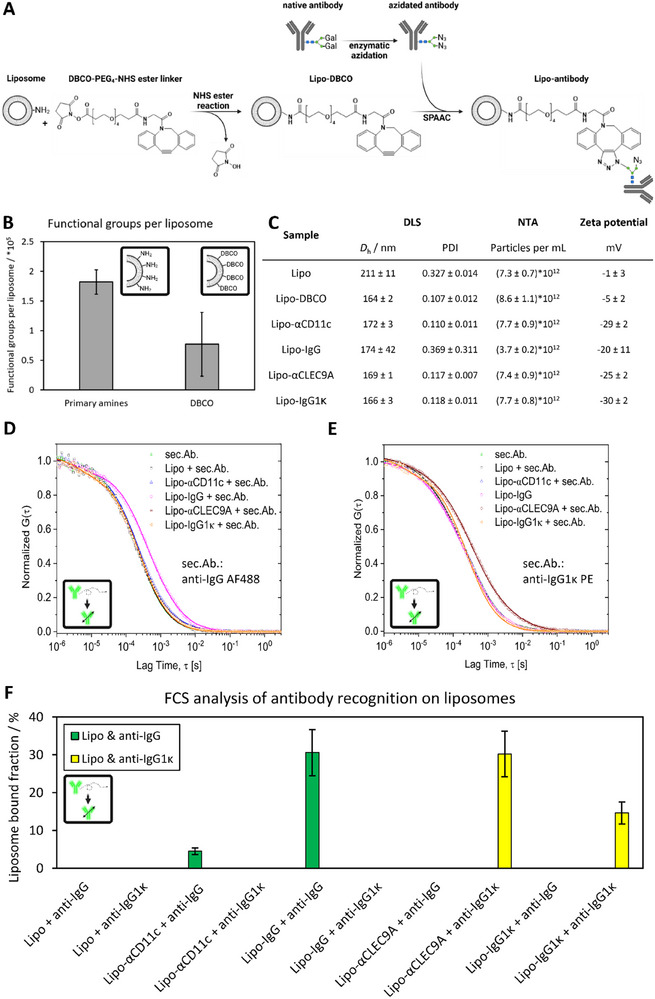
Liposome functionalization with antibodies and physicochemical characterization (batch for in vivo experiments). (A) Schematic summary of the liposome functionalization strategy. (B) Functional groups per liposome as detected via fluorescamine assay or anthracene azide assay. (C) DLS, NTA, and zeta potential analysis of liposome samples before and after antibody conjugation. (D) Autocorrelation curves from FCS analysis of liposomes with anti‐IgG AF488 secondary antibody. (E) Autocorrelation curves from FCS analysis of liposomes with anti‐IgG1κ PE secondary antibody. (F) Liposome‐bound fraction of secondary antibodies. Error bars represent an experimental error of ±10%. Created in BioRender. Schaaf, M. (2025) https://BioRender.com/vr1j9xi.

A schematic summary of the functionalization strategy is provided in Figure 2A. Antibodies against the DC surface receptors CD11c or CLEC9A, as well as their antibody isotype controls (IgG and IgG1κ, respectively), were employed in this system, creating four liposome batches with one antibody type each. Since liposome construct optimization in vitro and subsequent in vivo experiments both required large sample quantities, two separate liposome batches were produced.

Liposome functionalization was conducted in a two‐step process. First, a short PEG‐based linker that carried an N‐hydrosuccinimide (NHS)‐ester and a dibenzocyclooctyne (DBCO)‐group (strained alkyne) was covalently bound to the primary amines of DOPE lipids. Second, click chemistry was used to covalently attach the azidated antibody to the linker via the SPAAC click reaction. To ensure safe biological application, the potential unreacted azide was removed through three washing steps. The functionalization process was closely monitored through quantification of the relevant functional groups before and after DBCO‐linker conjugation to the liposomal surface, allowing for precise control over reactant ratios. Through assessment of various DBCO:NH_2_ and antibody:liposome (also expressed as DBCO:antibody) ratios, favorable conditions were identified and employed to achieve optimal targeting properties and colloidal stability. Figure 2B shows the functional groups per liposome as determined for the liposome batch used for in vivo experiments. Analysis via fluorescamine assay detected (1.8 ± 0.2)·10^5^ NH_2_ groups per liposome before linker conjugation, while analysis via anthracene azide assay detected (0.8 ± 0.5)·10^5^ DBCO groups per liposome after linker conjugation. These values imply that roughly 44% of available NH_2_ groups were conjugated to the DBCO‐containing linker, and roughly 59% of the total linker was conjugated to the liposomes (based on the initial DBCO:NH_2_ ratio of 0.75:1).

A range of antibody:liposome (Ab:Lipo) ratios was tested for each antibody type to determine the optimal ratio for in vivo experiments. To optimize the Ab:Lipo ratio, DBCO‐liposomes were functionalized with initial ratios of 20, 70, 140, or 280 Ab:Lipo. Basic characterization, including hydrodynamic diameter (*D*_h_) and polydispersity index (PDI), liposome concentration, and functional groups per liposome are provided in Figure S1.

For in vivo experiments, liposomes were functionalized with an initial ratio of 280 Ab:Lipo. Figure 2C provides a summary of basic characterization, including *D*_h_ and PDI, particle concentration, and zeta potential. Furthermore, multi‐angle dynamic light scattering (DLS) measurements in mouse plasma with multicomponent analysis were conducted to evaluate colloidal stability under physiological conditions [19]. When liposome constructs were incubated with mouse plasma at 37°C, no aggregation was detected for all samples, implying safe in vivo applicability (Figure S2). Corresponding *cryo* transmission electron microscopy (TEM) micrographs of exemplary liposome samples before and after functionalization are provided in Figure S3, showing that the functionalization procedure did not impair liposome morphology.

Overall, unfunctionalized and functionalized liposome samples of both the in vitro and in vivo batches showed similar parameters in hydrodynamic diameter *D*_h_, polydispersity index PDI, concentration, zeta potential, and number of functional groups within the usual small fluctuation between batches. The only exception was Lipo‐IgG, which appeared to be more polydisperse, indicated by the high PDI and larger variations in *D*_h_ and zeta potential. However, since no aggregation was detected in mouse plasma, the construct was considered as safe for in vivo use, although with additional measures such as a short centrifugation before use. The size and PDI decrease from Lipo to Lipo‐DBCO can be explained by the washing step after linker conjugation. In general, the batch‐to‐batch variation is rather small and mostly reflects in the DLS measurements as they are intensity‐weighted and, thus, very sensitive to even small concentrations of large aggregates. This may also be visible in zeta potential data to some extent, as the method relies on electrophoretic light scattering. Other physico‐chemical properties are generally less affected by batch effects.

To provide clear evidence for the respective antibody's presence at the liposomal surface, Lipo‐Abs were analyzed via FCS. For this purpose, Lipo‐Ab solutions were incubated with 10 equivalents (Ab:Lipo) of a fluorescently labeled secondary antibody against their respective surface‐bound antibody isotype (anti‐IgG secondary antibody against anti‐CD11c antibody and IgG isotype control antibody; anti‐IgG1κ secondary antibody against anti‐CLEC9A antibody and IgG1κ isotype control antibody). Subsequently, this mixture was analyzed via FCS.

Figure 2D,E shows FCS autocorrelation curves recorded for the fluorescent secondary antibodies before and after incubation with the liposomes. While Figure 2D shows samples that were incubated with IgG isotype‐detecting and Alexa Fluor 488 (AF488) labeled secondary antibody, Figure 2E shows samples that were incubated with IgG1κ isotype‐detecting and phycoerythrin (PE) labeled secondary antibody. Liposome‐bound secondary antibody fractions were then estimated by applying a two‐component fit to the FCS autocorrelation curves while keeping the sizes of the antibody and the liposome as fixed parameters (see Experimental Section for details). Deducted liposome bound secondary antibody fractions from all experiments are summarized in Figure 2F. Indeed, for functionalized samples, secondary antibody bound to the antibodies on the liposomal surface was detected, while for all samples serving as negative control, no binding was observed. This served as a qualitative confirmation that antibodies of the correct isotype are indeed present on and bound to the liposomal surface. Importantly, since fluorescence intensity scales differently for freely diffusing or liposome‐bound species, which can lead to overestimation of certain fractions, and fractions refer to secondary antibody‐bound liposomes, not primary antibody‐bound ones, quantitative information about the degree of liposome antibody functionalization cannot be deduced directly. Therefore, the information presented in Figure 2F should only be interpreted as a qualitative positive/negative assessment.

### 2.2 In Vitro Cell Uptake in Bone Marrow‐derived Dendritic Cells

To optimize DC subtype targeting, liposomes with different Ab:Lipo ratios were compared with regard to their uptake in target DCs in vitro.

**FIGURE 3:**
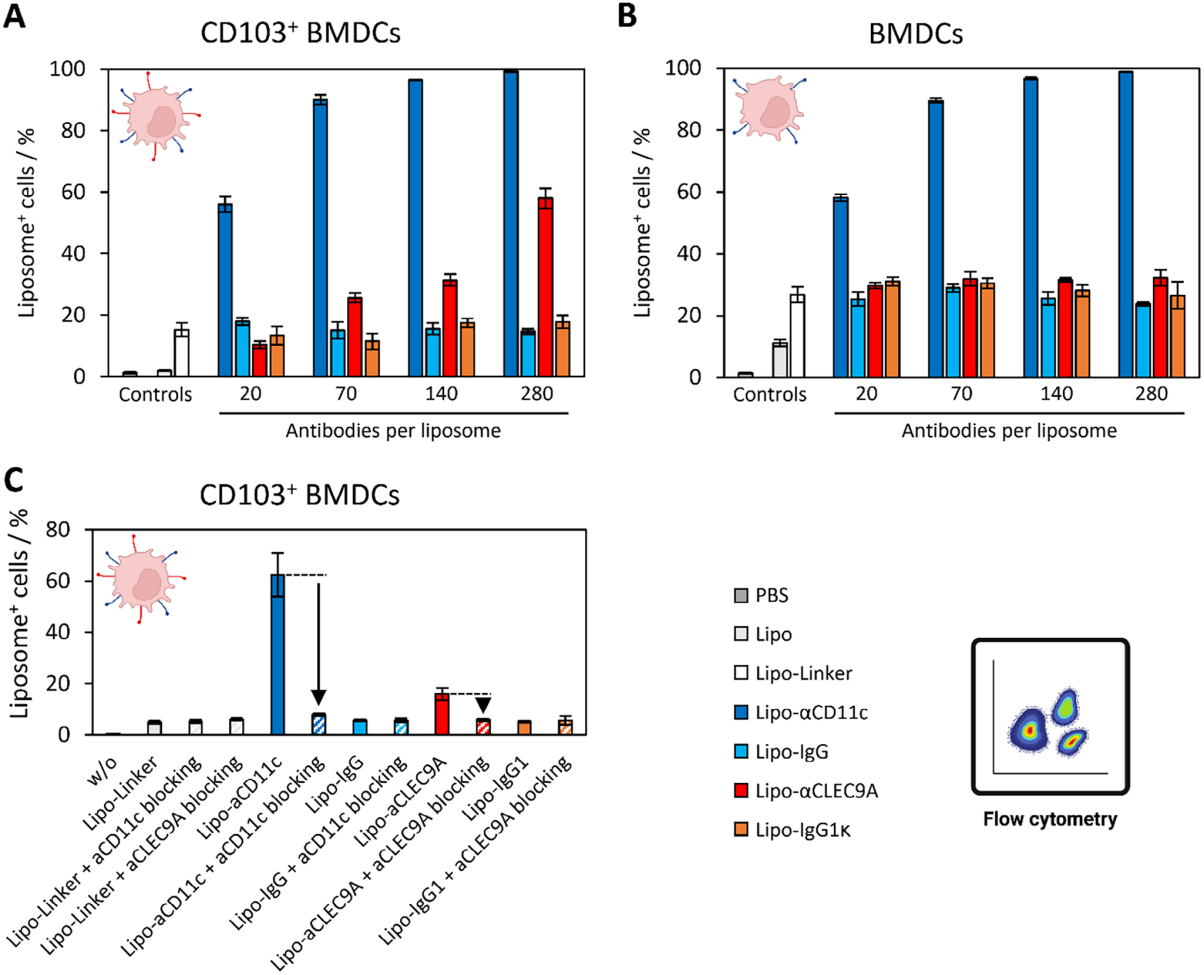
In vitro liposome cell uptake in regular or CD103^+^ BMDCs. Fraction of liposome‐positive cells. (A) Liposome cell uptake in CD103^+^ BMDCs (CD11c^+^, CLEC9A^+^). (B) Liposome cell uptake in regular BMDCs (CD11c^+^, CLEC9A^−^). (C) Liposome cell uptake after cell receptor blocking in CD103^+^ BMDCs. Values are the mean of three biological replicates ± standard deviation. Created in BioRender. Schaaf, M. (2025) https://BioRender.com/vr1j9xi.

Four different Ab:Lipo ratios (20, 70, 140, or 280) were employed during functionalization with each antibody type, resulting in a total of 16 Lipo‐Ab batches, including the isotype controls. To assess the effect of conjugated antibody type and/or amount on liposomal cell uptake, Lipo‐Abs were added to regular (granulocyte‐macrophage colony‐stimulating factor (GM‐CSF)‐derived) bone marrow‐derived dendritic cells (BMDCs) (CD11c^+^, CLEC9A^−^), or CLEC9A‐receptor expressing CD103^+^ BMDCs (CD11c^+^, CLEC9A^+^). According to the present surface receptors, anti‐CD11c antibody functionalization of liposomes should thus lead to increased cell uptake in both cell types, while anti‐CLEC9A functionalization should only have an effect on CD103^+^ BMDCs. Figure 3A shows the fraction of liposome‐positive CD103^+^ BMDCs, while Figure 3B shows the fraction of liposome‐positive regular BMDCs (fluorescence intensities shown in Figure S4A,B).

In both cell types, liposomes carrying anti‐CD11c antibody (Lipo‐αCD11c) showed a gradual increase in cell uptake, with complete saturation at 280 Ab:Lipo ∼99% liposome^+^ cells in regular BMDCs, and ∼99% liposome^+^ cells in CD103^+^ BMDCs. Similarly, liposomes carrying anti‐CLEC9A antibody (Lipo‐αCLEC9A) also showed a gradual increase in cell uptake in CD103^+^ BMDCs, although to a lesser extent than observed for Lipo‐αCD11c (Figure 3A). Here, the maximum was reached at ∼58% liposome^+^ cells when incubated with 280 Ab:Lipo Lipo‐αCLEC9A. Therefore, the Ab:Lipo ratio of 280 was determined to be most suitable for in vivo experiments. Since regular BMDCs do not express the CLEC9A‐receptor, no enhanced uptake of Lipo‐αCLEC9A was expected (Figure 3B).

Since an exact and direct determination of ligand density on the liposomal surface is not possible in a time‐efficient manner, a theoretical calculation based on several assumptions can be made. Assuming a liposome diameter of *D*_h_ = (175 ± 6) nm for the in vitro batch, an antibody diameter of 14 nm (for IgG antibodies), and conjugation of all available antibodies, the Ab:Lipo ratios of 20, 70, 140, or 280 yield roughly 3%, 11%, 22%, or 45% liposome surface area coverage, respectively. For the in vivo batch, assuming a liposome diameter of *D*_h_ = (164 ± 2) nm, the Ab:Lipo ratio 280 yields roughly 51% liposome surface coverage. While this represents only a rough estimation, it still shows that at the Ab:Lipo ratio of 280, a medium ligand density can be expected on the liposomal surface. In literature, medium ligand densities are often described as preferable for targeting, because they provide high avidity while maintaining low steric hindrance between targeting ligands [20, 21, 22, 23].

The observation that CD11c targeting led to a more pronounced enhancement of liposome cell uptake than CLEC9A targeting could be a result of the natural abundance of both receptors on the surface of DCs. Since CD11c is broadly expressed on several DC types and appears to play a more general role for DC function, it is likely to be more abundant than CLEC9A, which plays a more specialized role in the detection of necrotic cells [4, 6, 24]. Therefore, less enhanced cell uptake in the case of Lipo‐αCLEC9A could be a result of lower CLEC9A receptor abundance, rather than differences in antibody/receptor interactions. Reports on epidermal growth factor receptor (EGFR) targeting, as well as the known benefits of multiple receptor interactions, are in line with this theory [25, 26].

Additionally, receptor blocking experiments were conducted to evaluate the role of specific antibody/target interactions on liposomal cell uptake (Figure 3C, fluorescence intensities shown in Figure S4C). CD103^+^ BMDCs (CD11c^+^, CLEC9A^+^) were either directly incubated with Lipo‐Abs or pre‐incubated with free anti‐CD11c or anti‐CLEC9A antibody before liposome addition. By pre‐incubating cells with free antibody, the respective target receptors on the cellular surface (CD11c or CLEC9A) should be blocked, leaving no room for binding by liposome‐associated antibodies. If enhanced liposomal cell uptake can be decreased to antibody isotype control‐levels through receptor blocking, this implies that the increased uptake was specific [27, 28]. As shown in Figure 3C, cell uptake decreases from an elevated uptake to the baseline level could be observed for Lipo‐αCD11c (from ∼62% to ∼8% liposome^+^ cells) and Lipo‐αCLEC9A (from ∼16% to ∼6% liposome^+^ cells). Therefore, it was concluded that enhanced cell uptake of Lipo‐αCD11c or Lipo‐αCLEC9A constructs in CD103^+^ BMDCs (CD11c^+^, CLEC9A^+^) was mediated by specific antibody/receptor interactions.

### 2.3 Ex Vivo Cell Uptake in Non‐Parenchymal Cells

After confirming enhanced antibody‐specific uptake of Lipo‐αCD11c and Lipo‐αCLEC9A in target‐receptor expressing BMDCs, we aimed for cell systems that are closer to in vivo conditions. Therefore, non‐parenchymal cells (NPCs) were extracted from the mouse liver and incubated with liposomes ex vivo. The resulting cell uptake graphs are summarized in Figures S5 and S6. Analyzed NPC cell types include cDC1, cDC2, plasmacytoid DC (pDC) subtype DCs, Kupffer cells, and liver sinusoidal endothelial cells (LSECs).

Lipo‐αCD11c showed enhanced uptake in cDC1s, cDC2s, and pDCs, with a similar trend of a more pronounced uptake with higher antibody density, as previously observed in BMDCs. Lipo‐αCLEC9A did not show enhanced uptake in cDC1s, and only slightly enhanced uptake at 280 Ab:Lipo in pDCs. Kupffer cells and LSECs showed high cell uptake for all linker‐ and/or antibody‐functionalized liposome variants. Due to the natural scavenging function of these cell types, this is expected and must be attributed to unspecific cell uptake [29, 30, 31].

Due to the large number of analyses and limitations in batch sizes, only one biological replicate could be performed in this analysis. However, since the aim of this analysis was merely the confirmation of previous in vitro results and a first approximation of in vivo conditions, this was tolerated.

### 2.4 In Vivo Biodistribution

**FIGURE 4:**
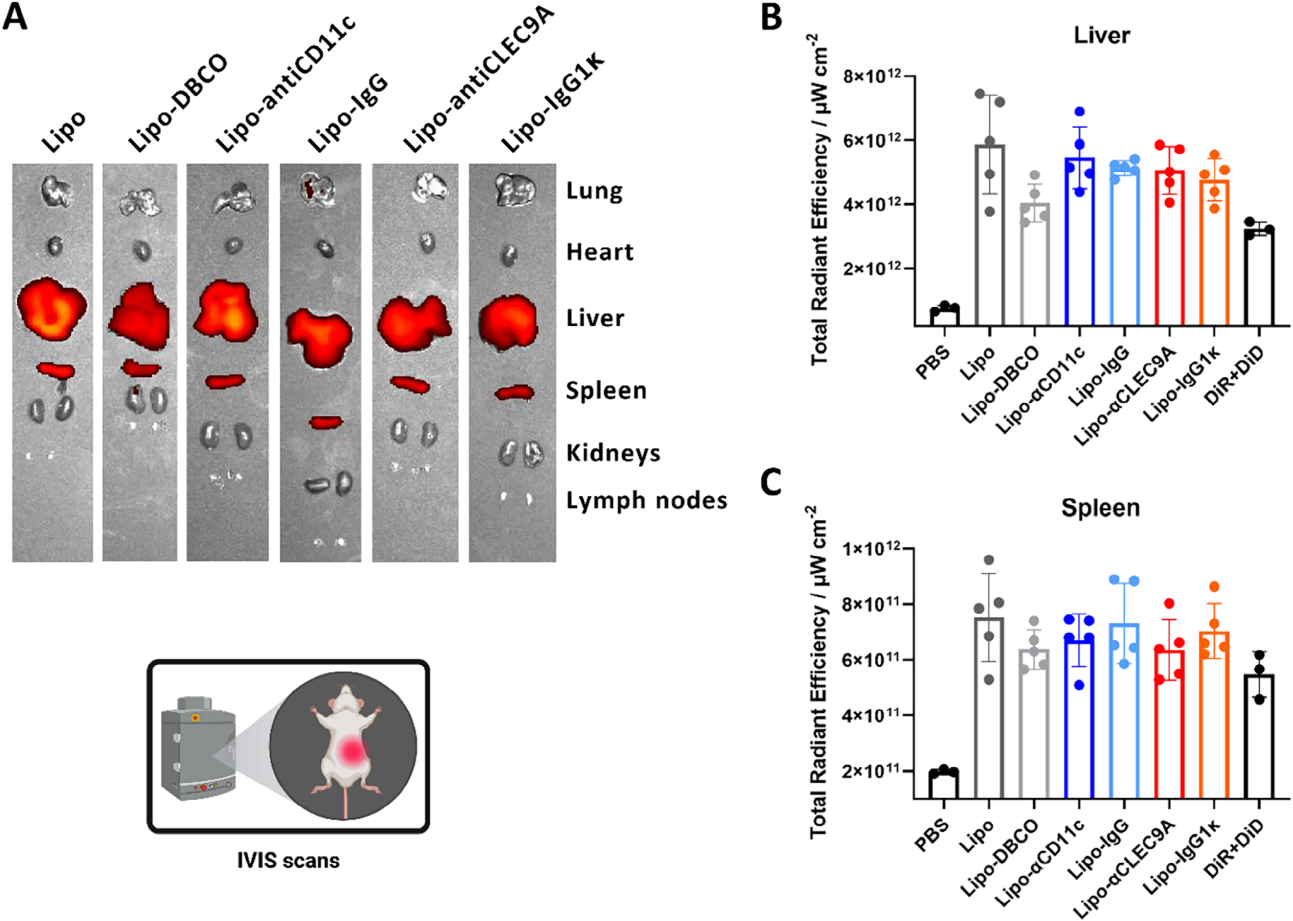
In vivo liposome biodistribution in animals injected with different liposome constructs. (A) IVIS scans of organs. (B) IVIS scan total radiant efficiency of livers. (C) IVIS scan total radiant efficiency of the spleens. Values are the mean of five biological replicates (three for PBS and DiD+DiR controls) ± standard deviation. Created in BioRender. Schaaf, M. (2025) https://BioRender.com/vr1j9xi.

Based on promising cell targeting results in vitro and ex vivo, in vivo experiments were carried out as the final step. Mice were injected intravenously with liposome constructs that carried two membrane dyes: DiD for flow cytometry and DiR for in vivo imaging system (IVIS) scans. After incubation, mice were sacrificed, and organs were analyzed via IVIS scans to determine the liposome biodistribution. To address biocompatibility, we performed live/dead staining of all cells following treatment (see Figures S10 and S11). The analysis showed no significant differences in viability between liposome‐treated groups and PBS control. However, it has to be noted that the data presented is only showing the specific effect on the target cells, while organ‐level toxicity, that is, by using blood chemistry and histopathological analysis, was not investigated. Figure 4A shows a comparison between organ scans of mice incubated with different liposome constructs (all animals shown in Figure S7). Only liver and spleen showed an elevated fluorescence signal intensity, implying that liposome accumulation mostly occurred in these two organs, which is expected for nanoparticles of this size range [32, 33, 34].

Furthermore, low signal in the lung verified the absence of major aggregates as indicated by multiangle DLS, which otherwise would have accumulated in the fine capillaries. Also, a low signal in the heart and lymph nodes implied a low concentration of liposomes in the blood circulation and the lymphatic system.

More detailed comparisons between liposome constructs by quantifying the fluorescence signal intensity (Figure 4B,C, whole animal and all organs shown in Figure S8) showed no significant difference between the constructs. Since liposomes with and without a linker, and with and without antibodies, showed no difference in biodistribution, it can be assumed that accumulation in the liver and spleen was probably due to nanocarrier size and not surface properties [32, 33, 34].

### 2.5 In Vivo Cell Uptake in Non‐Parenchymal Cells and Splenocytes

**FIGURE 5:**
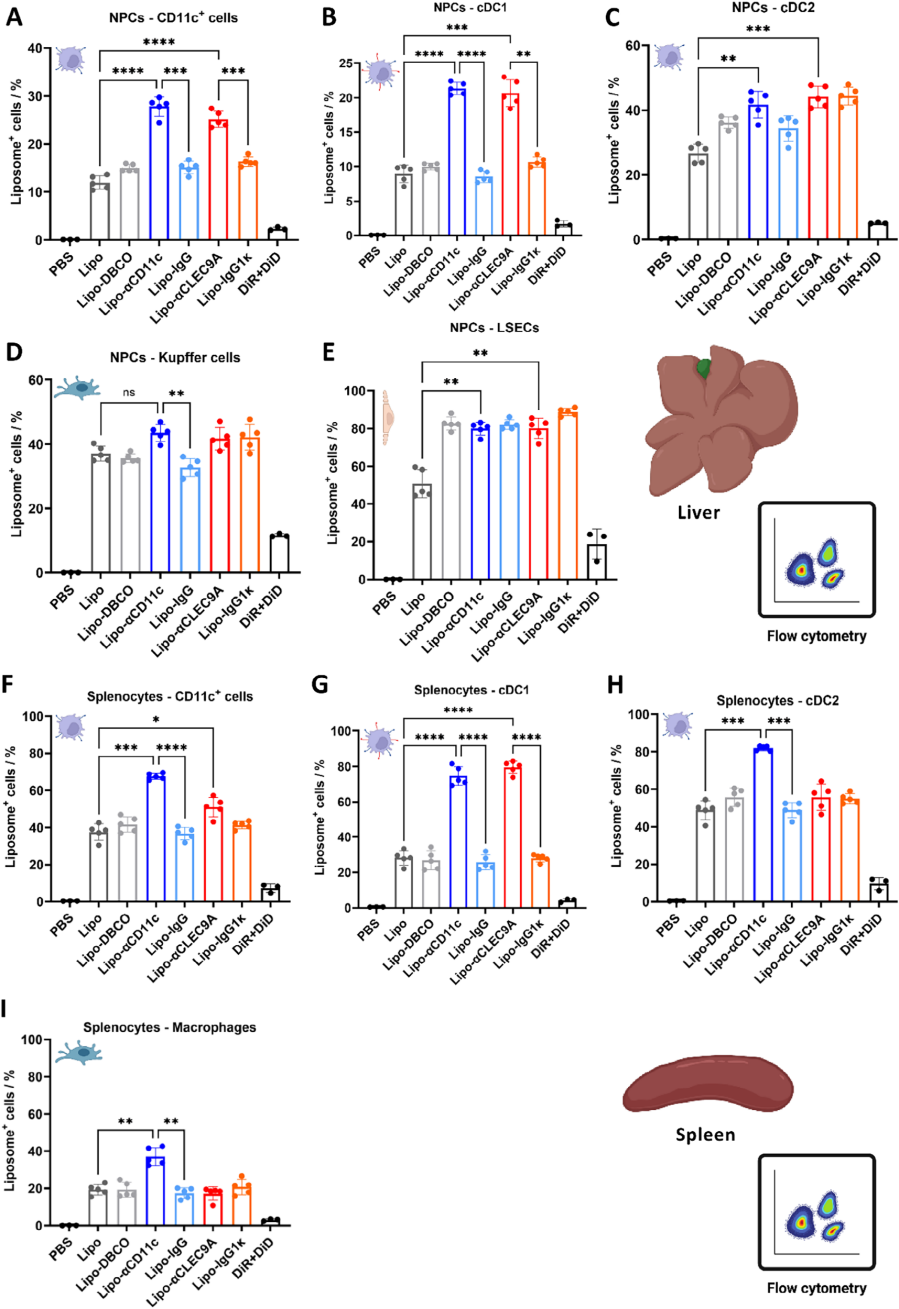
In vivo liposome cell uptake in liver and spleen. Fraction of liposome‐positive cells. (A) CD11c^+^ NPCs. (B) cDC1 NPCs. (C) cDC2 NPCs. (D) Kupffer cell NPCs. (E) LSEC NPCs. (F) CD11c^+^ splenocytes. (G) cDC1 splenocytes. (H) cDC2 splenocytes. (I) Macrophage splenocytes. Values are the mean of five biological replicates (three for PBS and DiD+DiR controls) ± standard deviation. Significance levels were determined through Brown‐Forsythe and Welch ANOVA tests; asterisks indicate the following *p‐values*: ^*^ *p* < 0.05, ^**^ *p* < 0.01, ^***^ *p* < 0.001, ^****^ *p* < 0.0001, ns = not significant. Created in BioRender. Schaaf, M. (2025) https://BioRender.com/vr1j9xi.

After distribution evaluation at the organ level, the next step was detailed flow cytometric analyses of uptake in different hepatic and splenic immune cells of the collected organs to determine liposome localization on the cellular level. Figure 5A–E shows flow cytometric analyses of NPCs (fluorescence intensities shown in Figure S9). Here, cDC1 type cells showed significantly enhanced uptake of Lipo‐αCD11c (∼21% liposome^+^ cells) and Lipo‐αCLEC9A (∼21% liposome^+^ cells) liposome constructs when compared to the antibody isotype controls (∼9% liposome^+^ cells for Lipo‐IgG and ∼11% liposome^+^ cells for Lipo‐IgG1κ). In cDC2 type cells, Kupffer cells, and LSECs, Lipo‐αCD11c and Lipo‐αCLEC9A uptake showed no significant difference to the antibody isotype controls. In comparison to observations in vitro, the unfunctionalized control Lipo showed an elevated cell uptake in vivo. This is probably a result of the more complex biological microenvironment the liposome encounters in vivo, with the presence of serum proteins promoting the formation of a protein corona, a different cell microenvironment, phenotypes, and activation states.

In splenocytes (Figure 5F–J), cDC1 type cells showed significantly enhanced uptake of Lipo‐αCD11c (∼75% liposome^+^ cells) and Lipo‐αCLEC9A (∼80% liposome^+^ cells) liposome constructs when compared to the antibody isotype controls (∼26% liposome^+^ cells for Lipo‐IgG and ∼28% liposome^+^ cells for Lipo‐IgG1κ). Also, cDC2 type cells showed significantly enhanced uptake of Lipo‐αCD11c (∼82% liposome^+^ cells) liposome constructs when compared to Lipo‐IgG1κ (∼49% liposome^+^ cells).

In short, a significant enhancement of liposome uptake in cDC1 cells could be observed in both liver and spleen. Based on the expected in vivo biodistribution (accumulation in liver and spleen), which was confirmed by IVIS scans (Figure 4A), and the observed enhanced uptake into CLEC9A‐expressing BMDCs in vitro (Figure 3A), this enhanced liposome uptake in cDC1 cells coincided with our expectations and confirmed that successful targeting could be transferred from in vitro to in vivo settings. Despite high uptake into tissues and cell types other than the target cells, which is a commonly observed phenomenon in drug targeting, incrementally increasing the share of carriers that reach the target cells by active targeting—even if only by a few percent—can enhance a treatment‘s efficacy significantly. It means lower overall doses are required to reach the same dose at the target site, and an increased percentage reaching the target also decreases the amount of drug in off‐target sites, lowering the potential for side effects. In the case of Lipo‐CLEC9A, the drug carrier amount per injection (and, thus, potentially the amount of active ingredient) required to reach an equal effect in hepatic (NPC) cDC1 cells (Figure 5B) would roughly be half of the dose used in untargeted approaches (Lipo or Lipo‐IgG1κ, estimation based on the percentage of liposome‐positive cells). Similarly, to reach an equal effect in splenic cDC1 cells (Figure 5G) it would roughly be one third. To increase patient safety and decrease costs, reducing the drug dose by half or two‐thirds would provide massive benefits, leading both to a lower risk of side effects and lower material costs for drugs.

The following studies could include the introduction of immunostimulatory agents into the liposomal carrier system to trigger cDC1 activation, leading to a protective T cell response. To ensure the development of target immunity instead of tolerance, liposomes could be loaded with a combination of a stimulatory adjuvant and a target‐specific antigen.

## 3 Conclusions

In this study, dendritic cell targeting antibodies were conjugated to liposomes to achieve active targeting toward splenic and hepatic cDC1 cells in vivo. Through multifaceted physicochemical analyses and profound in vitro studies, the liposomal constructs were thoroughly characterized and optimized toward high target specificity.

The antibody density on the liposomal surface was optimized by combining information from functional group quantification assays and in vitro cell uptake experiments in BMDCs. Ex vivo cell uptake experiments in liver NPCs were used to approximate in vivo conditions while maintaining a large number of samples and antibody densities. They were used to confirm our findings from single‐cell type in vitro experiments by increasing complexity to a mix containing multiple cell types, and served as a first indicator for successful in vivo application. The antibody attachment to the liposomal surface was confirmed through FCS, and multiangle DLS in mouse plasma provided a quality control checkpoint to assure safe in vivo application. Liposome size and surface charge, the main determinants for passive targeting, were determined through DLS and zeta potential measurements, and their effect on the liposomes‘ in vivo biodistribution was observed through IVIS scans. Based on the observed liposome accumulation in liver and spleen, flow cytometric analyses of hepatic and splenic tissues provided a more detailed quantitative analysis of various cell types and subtypes.

As a result, a strong increase in hepatic and splenic cDC1 subtype‐specific cell uptake was observed for the final anti‐CLEC9A antibody conjugated construct. To the best of our knowledge, these results show the highest enhancement of cDC1‐specific liposome uptake reported for an in vivo system so far. Through the combination of the superior target specificity of anti‐CLEC9A antibody with a highly established nanocarrier system presented by liposomes, this approach provides a powerful platform for a clinical translation of highly specific immunotherapeutic applications.

## 4 Experimental Section

### 4.1 Materials

All chemicals and materials were used as received. Acetone (≥ 99.8% purity) was obtained from VWR (Germany). Ampuwa water was obtained from Fresenius Kabi (Germany). 1,2‐Dioleoyl‐*sn*‐glycero‐3‐phosphoethanolamine (DOPE, 10 mg mL^−1^, in chloroform, ≥ 99.0% purity) and L‐α‐phosphatidylcholine (egg PC, 25 mg mL^−1^, in chloroform, ≥ 99.0% purity) were obtained from Avanti Research (USA). 1,1'‐Dioctadecyl‐3,3,3',3'‐tetramethylindodicarbocyanine perchlorate (DiD), dimethyl sulfoxide (DMSO, 99.7+% purity), fluorescamine, Pierce 660 nm Protein Assay Reagent, mouse anti‐rat IgG1 secondary antibody, PE, eBioscience (clone R1‐12D10, 0.2 mg mL^−1^), and Site Click Antibody Azido Modification Kit were obtained from Thermo Fisher Scientific (Germany). Chloroform, cholesterol (Chol), phosphate‐buffered saline (PBS, ‐Mg^2+^, ‐Ca^2+^) (product number D8537), and bovine serum albumin (BSA, ≥ 98% purity) were obtained from Sigma Aldrich (Germany). DBCO‐PEG_4_‐NHS ester linker was obtained from Jena Bioscience (Germany). 1,1'‐Dioctadecyl‐3,3,3',3'‐tetramethylindotricarbocyanine iodide (DiR) was obtained from Abcam (UK). Di‐sodium tetraborate decahydrate (borax) was obtained from Merck Millipore (Germany). Glycine was obtained from Carl Roth (Germany). Anthracene azide (Anth‐N_3_) was synthesized in‐house according to a previously published procedure [35]. Anti‐mouse CD11c antibody (clone N418, 0.5 mg mL^−1^), purified armenian hamster IgG isotype control antibody (clone HTK888, 0.5 mg mL^−1^), purified rat IgG1 κ isotype control antibody (clone RTK2071, 0.5 mg mL^−1^), and Alexa Fluor 488 goat anti‐hamster (armenian) IgG antibody (clone Poly4055, 0.5 mg mL^−1^) were obtained from Biolegend (USA). Anti‐mouse CLEC9A antibody (clone 7H11, 8.34 mg mL^−1^) was obtained from BioXCell (USA).

### 4.2 Methods

#### 4.2.1 Liposome Production and Functionalization

##### 4.2.1.1 Liposome Production

Liposomes were produced via the lipid film hydration method followed by extrusion through a set of membranes according to a previously published procedure [14]. Briefly, a lipid mixture consisting of L‐α‐phosphatidylcholine (eggPC), 1,2‐dioleoyl‐*sn*‐glycero‐3‐phosphoethanolamine (DOPE), and cholesterol (Chol) in the molar ratio of 1:1:1 and a total lipid content of 30 µmol was dissolved in a total of 3.5 mL chloroform in a 50 mL round‐bottom flask. 30 nmol DiD (26 mM stock solution in DMSO) was added for a final content of 0.1 mol% related to total lipids. In the batch produced for in vivo experiments, DiR was added at the same concentration as DiD. The flask was attached to a rotary evaporator, and the chloroform was evaporated at 350 mbar, 40°C, and 150 rpm for 30 min. Residual chloroform was evaporated in a second step at 10 mbar, 40°C, and 150 rpm for 1 h. The lipid thin film was then hydrated through the addition of 4 mL PBS and vortexing until the film was completely detached from the glass. The mixture was stirred with a magnetic stirrer at room temperature (RT) and 500 rpm overnight. Afterwards, the solution was sonicated in a water bath for 20 min at RT and then extruded through polycarbonate membranes to obtain small unilamellar liposomes. The membrane pore sizes were 800, 400, 200, and 100 nm, with eleven extrusions through each membrane. The final liposome solution was stored at 4°C in the dark until further use.

##### 4.2.1.2 Quantification of Primary Amine Groups on the Surface of Liposomes (Fluorescamine Assay)

To quantify the primary amines on the liposomes’ surface, primary amine groups were derivatized through the addition of fluorescamine, followed by fluorescence readout. Standard solutions containing 1, 0.7, 0.5, 0.2, 0.1, 0.05, or 0 mM glycine were used to establish a calibration curve for primary amine concentration. In a 1.5 mL Eppendorf tube, 725 µL borate buffer (0.1 M, pH 9.5) were added to 25 µL standard or sample solution. 250 µL fluorescamine solution (0.3 mg mL^−1^, in acetone) were added, followed by vortexing at maximum speed for 30 s. After that, 100 µL of the mix was pipetted immediately into three wells of a black, clear bottom 96 well‐plate. The fluorescence signal was recorded at 25°C via a plate reader, with *λ*_Ex_ = 410 nm and *λ*_Em_ = 470 nm. Each sample was measured at 0.5x, 0.25x, or 0.125x concentration, and in technical triplicates. Only concentrations that showed values within the linear range of the glycine standard were considered, and their deduced values for 1x concentration were averaged.

##### 4.2.1.3 Liposome Surface Functionalization with DBCO‐PEG_4_‐NHS Ester Linker

Primary amines on the liposomal surface were subsequently functionalized with DBCO‐PEG_4_‐NHS ester linker at a molar ratio of 0.75:1 (DBCO:NH_2_).

Batch for in vitro experiments

In a 4 mL glass vial, 49.20 µL linker stock solution (61.57 mM, in DMSO) was added to 2 mL liposome solution (0.42 wt.% solid content, 2.02 mM primary amines), followed by vortexing for 5 s and an incubation at RT and 500 rpm in a thermoshaker for 2 h in the dark.

Batch for in vivo experiments

In a 4 mL glass vial, 67.20 µL linker stock solution (61.57 mM, in DMSO) was added to 2.5 mL liposome solution (0.29 wt.% solid content, 2.21 mM primary amines), followed by vortexing for 5 s and an incubation at RT and 500 rpm in a thermoshaker for 2.5 h in the dark.

To remove excess linker, the DBCO‐liposome solution was subjected to three washing steps. Each washing step consisted of centrifugation in pre‐wetted Amicon filters (100 kDa MWCO) at RT and 3,000 g for 1 h. After discarding the flow‐through, the supernatant was filled up to 500 µL with PBS. After completing the third washing step, the supernatant was recovered by inverting the filter into a fresh tube and centrifugation at RT and 3,000 g for 10 min. Recovered supernatants were pooled, and PBS was added to reach the initial volume of the DBCO‐liposome solution.

##### 4.2.1.4 Quantification of DBCO Groups on the Surface of Liposomes (Anthracene Azide Assay)

To quantify the amount of DBCO groups on the liposomal surface, an anthracene azide (Anth‐N_3_) assay was performed according to a previously published procedure [35]. An Anth‐N_3_ stock solution (1.7 mg mL^−1^ in DMSO) was prepared immediately before the assay and covered with aluminum foil. The following sub‐samples were prepared: (a, b) 32.6 µL DMSO + 17.3 µL Anth‐N_3_ stock solution, (c, d) 7.63 µL DMSO + 17.3 µL Anth‐N_3_ stock solution + 25 µL liposome sample, (e) 25 µL DMSO + 25 µL liposome sample. All samples were vortexed, covered with aluminum foil, and incubated on a stirring plate at RT and 300 rpm overnight. On the following day, each sub‐sample (a‐e) was used to prepare 100 µL of a 10x and a 100x dilution in DMSO. Each of those diluted sub‐samples was transferred to a black, clear bottom 96 well‐plate. The fluorescence signal was recorded at 25°C via a plate reader, with *λ*_Ex_ = 370 nm and *λ*_Em_ = 414 nm. The measurement data were processed according to a previously described procedure [15].

##### 4.2.1.5 Site‐Specific Enzymatic Antibody Azidation and Pierce Assay

Four different antibody types were enzymatically azidated:

Anti‐mouse CD11c antibody (clone N418, Biolegend, 0.5 mg mL^−1^), purified armenian hamster IgG isotype control antibody (clone HTK888, Biolegend, 0.5 mg mL^−1^), anti‐mouse CLEC9A antibody (clone 7H11, BioXCell, 8.34 mg mL^−1^), and purified rat IgG1 κ isotype control antibody (clone RTK2071, Biolegend, 0.5 mg mL^−1^). 300 µg of each antibody was site‐specifically azidated via the Site Click Antibody Azido Modification Kit (Thermo Fisher), following the kit protocol.

After completion of the azidation protocol, the concentration of azido‐antibodies was determined through a Pierce assay. Standard solutions containing 2 mg mL^−1^, 1 mg mL^−1^, 500, 250, 125, 62.5, or 31.25 µg mL^−1^ bovine serum albumin (BSA) in water were used to establish a calibration curve for protein concentration. For azido‐antibody samples, 10 µL of a 0.5x concentration (5 µL azido‐antibody solution + 5 µL water) and 10 µL of a 0.25x concentration (2.5 µL azido‐antibody solution + 7.5 µL water) were used. For the assay, 10 µL of standard or diluted sample was pipetted into the wells of a transparent 96‐well plate. 150 µL Pierce reagent was added, and the plate was incubated at RT for 5 min in the dark. After incubation, the plate was immediately transferred to the plate reader, where absorbance at 660 nm was recorded at 25°C.

After determining their protein concentration via Pierce assay, azido‐antibody solutions were stored at 4°C in the dark until further use.

##### 4.2.1.6 Liposome Surface Functionalization with Antibodies

Liposome surface functionalization with defined antibody‐per‐liposome (Ab:Lipo) amount was based on the following calculations and assumptions:

First, liposome concentration (liposome number mL^−1^) in unfunctionalized liposome solutions was calculated based on average hydrodynamic diameter and solid content. This estimation was chosen because exact concentration determination via nanoparticle tracking analysis (NTA) was not available at all times. Second, liposome concentration was assumed to remain constant throughout linker conjugation.

Batch for in vitro experiments

To optimize the Ab:Lipo ratio, four different ratios (20:1, 70:1, 140:1, 280:1 (Ab:Lipo)) were tested for each antibody.

Based on the estimated DBCO‐liposome concentration (6.57·10^12^ liposomes mL^−1^), the results from Anth‐N_3_ assay (3.15·10^4^ DBCO per liposome) and Pierce assay (*c*_azido‐αCD11c_ = 2.40 mg mL^−1^, *c*_azido‐IgG_ = 1.98 mg mL^−1^, *c*_azido‐αCLEC9A_ = 3.10 mg mL^−1^, *c*_azido‐IgG1κ_ = 2.61 mg mL^−1^), the azido‐antibody volumes were used for functionalization of 100 µL DBCO‐liposome solution as displayed in Table 1.

**TABLE 1:** Azido‐antibody solution volumes required for liposome functionalization.

Azido‐antibody	Azido‐antibody volume (µL) for an Ab:Lipo ratio of			
	20:1	70:1	140:1	280:1
azido‐αCD11c	1.4	4.8	9.5	19.1
azido‐IgG	1.7	5.8	11.6	23.1
azido‐αCLEC9A	1.1	3.7	7.4	14.8
azido‐IgG1κ	1.3	4.4	8.8	17.6

Here, 20:1 (Ab:Lipo) = 1575:1 (DBCO:Ab), 70:1 (Ab:Lipo) = 450:1 (DBCO:Ab), 140:1 (Ab:Lipo) = 225:1 (DBCO:Ab), and 280:1 (Ab:Lipo) = 113:1 (DBCO:Ab).

Batch for in vivo experiments

For in vivo experiments, only one ratio (280:1 (Ab:Lipo)) was used for each antibody.

Based on the estimated DBCO‐liposome concentration (6.06·10^12^ liposomes mL^−1^), the results from Anth‐N_3_ assay (1.09·10^5^ DBCO per liposome) and Pierce assay (*c*_azido‐αCD11c_ = 0.86 mg mL^−1^, *c*_azido‐IgG_ = 0.93 mg mL^−1^, *c*_azido‐αCLEC9A_ = 1.11 mg mL^−1^, *c*_azido‐IgG1κ_ = 1.07 mg mL^−1^), the following azido‐antibody volumes were calculated for 400 µL DBCO‐liposome solution:

Azido‐antibody volume for an Ab:Lipo ratio of (280:1): 197 µL azido‐αCD11c, 181 µL azido‐IgG, 152 µL azido‐αCLEC9A, 158 µL azido‐ IgG1κ. Here, 280:1 (Ab:Lipo) = 391:1 (DBCO:Ab).

In 1.5 mL Eppendorf tubes, DBCO‐liposomes were mixed with the respective azido‐antibody volumes, pipetted up and down, and incubated at RT and 500 rpm in a thermoshaker for 17 h in the dark. To remove excess azido‐antibodies, the liposome solution was subjected to three washing steps. Each washing step consisted of centrifugation in pre‐wetted Amicon filters (300 kDa MWCO) at RT and 3,000 g for 45 min. After discarding the flow‐through, the supernatant was filled up with PBS. After completing the third washing step, the supernatant was recovered by inverting the filter into a fresh tube and centrifugation at RT and 3,000 g for 10 min. PBS was added to reach the initial volume of each sample. Solutions were stored at 4°C in the dark until further use.

#### 4.2.2 Physicochemical Characterization

##### 4.2.2.1 Solid Content and Estimation of Liposome Concentration

To determine the solid content of unfunctionalized liposome solutions, the solvent was evaporated, and the dry mass was determined relative to a PBS blank. To estimate liposome concentration based on average hydrodynamic diameter and solid content, calculations were performed as previously described by Mateos–Maroto et al. [14].

##### 4.2.2.2 Dynamic Light Scattering (DLS)

The hydrodynamic diameter distribution and polydispersity index (PDI) of liposome solutions were measured at 25°C in a total volume of 200 µL (10 µL sample + 190 µL PBS), using a Zetasizer Nano S90 (Malvern Panalytical GmbH, Germany). Reported values are the mean of Z‐average values of the main peaks of three technical replicates ± standard deviation.

##### 4.2.2.3 Multi‐Angle DLS

Multi‐angle DLS experiments were performed on a commercially available instrument from ALV GmbH (Germany) consisting of an electronically controlled goniometer and an ALV‐5000 multiple tau full‐digital correlator. A He/Ne laser with a wavelength of 632.8 nm and output power of 25 mW (JDS Uniphase, USA, Type 1145P) was utilized as the light source. Measurements were performed at 37°C at seven angles ranging from 30° to 150°. 50 µL liposome solution (0.6 mg mL^−1^, filtered through a 0.45 µm Millex‐LG filter) was added to 200 µL mouse plasma (filtered through a 0.2 µm Millex‐GS filter) and incubated before measurement for 1 h at 37°C.

Reference measurements needed for the evaluation were performed with 50 µL liposome solution (0.6 mg mL^−1^) in 200 µL PBS after being filtered through a 0.45 µm Millex‐LG filter. 200 µL mouse plasma was diluted with 50 µL PBS and filtered through a 0.2 µm Millex‐GS filter. For data analysis, a method previously described by Rausch et al. was applied [19]. Mouse plasma was obtained from 6–12 week‐old C57BL/6J and C57BL/6 albino mice (see section Biological Evaluation).

##### 4.2.2.4 Zeta Potential

Zeta potential of liposome solutions was measured at 25°C in a total volume of 800 µL (10 µL sample + 790 µL 0.1 M KCl), using a Zetasizer Nano Z (Malvern Panalytical GmbH, Germany). Zeta potential values were calculated as averages and standard deviation of the main peaks of three technical replicates.

##### 4.2.2.5 Nanoparticle Tracking Analysis (NTA)

Nanoparticle tracking analysis was used to determine liposome concentration in solution for liposomes used for in vivo experiments. Particles were measured using a ZetaView Nanoparticle Tracking Analyzer PMX‐230 (TWIN) (Particle Metrix, Germany). Samples were diluted 20,000x, 50,000x, or 100,000x before measurement. Data were analysed using the ZetaView software (version 8.05.16 SP3). Particle movement was recorded in scatter mode with 1 cycle at 11 positions, at 25°C.

##### 4.2.2.6 Fluorescence Correlation Spectroscopy (FCS)

FCS experiments were performed using a commercial confocal microscope, LSM 880 (Carl Zeiss, Jena, Germany) equipped with a C‐Apochromat 40×/1.2 W (Carl Zeiss, Jena, Germany) water immersion objective. An Argon‐Ion laser (*λ *= 488 nm) and a HeNe laser (*λ *= 543, 633 nm) fiber coupled to the LSM 880 were used for the excitation of the Alexa Fluor 488‐labeled secondary antibodies, PE‐labeled secondary antibodies, and DiD‐labeled liposomes, respectively. The emission light at 500 – 553 nm, 565 – 610 nm, and 655 – 700 nm was detected using a spectral detection unit (Quasar, Carl Zeiss). For each sample, 200 µL of the solution was added to the 8‐well polystyrene‐chambered cover glass (Nunc Lab‐Tek, Thermo Fisher Scientific, Waltham, MA). The confocal detection volume was positioned 90 µm above the glass coverslip, and a series of 20 measurements, 10 s each, was performed at room temperature (23°C). The obtained experimental autocorrelation curves were fitted with the following analytical model function:

(1) $$G\left(\tau \right)=1+\left[1+\frac{f_{T}}{1-f_{T}}e^{-\tau /\tau_{T}}\right]\frac{1}{N}\sum_{i=1}^{m}\frac{f_{i}}{\left(1+\frac{\tau }{\tau_{D,i}}\right)\cdot \sqrt{1+\frac{\tau }{S^{2}\cdot \tau_{D,i}}}}$$

whereby *f_T_* and *τ_T_* are the fraction and the decay time of the triplet state, *N* is the average number of diffusing fluorescence species in the observation volume, τ_D_ *_i_* is the diffusion time of the *i‐*th type of species, *f_i_* is the fraction of component *i* (1 ≤ *i* ≤ *m*), and *S* is the structure parameter, *S* = *z*_0_/*r*_0_, where *z*_0_ and *r*_0_ represent the axial and radial dimensions of the observation volume. The fits were done using the ZEN 3.0 software (Carl Zeiss, Jena, Germany), which yielded the values of *τ_Di_* , *N*, and *S*. The diffusion coefficients of the species *D_i_* are related to the respective diffusion times *τ_Di_* and the radial dimension *r*_0_ of *V*_obs_ by *D_i_ = r_0_^2^/(4τ_Di_)*. By inserting *D_i_* into the Stokes‐Einstein equation, the hydrodynamic radius can be calculated as $R_{h}=\frac{k_{B}\cdot T}{6\cdot \pi \cdot \eta \cdot D}\;$ Here, *k*_B_ is the Boltzmann constant, *T* is the temperature, and *η* is the viscosity of the solvent. For solutions containing only labeled secondary antibodies or only DiD‐labeled liposomes, single‐component (m = 1) fits were applied that yielded the diffusion times, the diffusion coefficients, and the sizes of the antibodies and the liposomes. For solutions of labeled secondary antibodies incubated with liposomes, a two‐component fit (Equation 1 with m = 2) was applied in which the previously determined diffusion times of the two components (antibodies and liposomes) were kept as fixed parameters. The fits yielded the fractions, *f_i_* of the two components, and thus the liposome‐bound secondary antibody fraction.

The calibration of the confocal volume *V*_obs_ in each channel was performed by independent FCS experiments with reference dyes with known diffusion coefficients, namely Alexa Fluor 488, Alexa Fluor 546, and Atto 643.

##### 4.2.2.7 Cryogenic Transmission Electron Microscopy (cryo‐TEM)

For *cryo*‐TEM examination, the samples were vitrified using a Vitrobot Mark V (Thermo Fisher, Hilsboro Oregon) plunging device. 3 µL of the sample dispersion was applied to a Quantifoil or a lacey carbon‐coated TEM grid that was glow‐discharged in an oxygen plasma cleaner (Diener Nano, Diener electronic, Germany) shortly before. After removing excess sample solution with a filter paper, the grid was immediately plunged into liquid ethane. For the subsequent examination, the specimen was transferred to a TEM (FEI Titan Krios G4), maintaining cryogenic conditions.

Conventional TEM imaging was done using an acceleration voltage of 300 kV. Micrographs were acquired with a 4k Direct Electron Detection Camera (Gatan K3) under low‐dose conditions.

#### 4.2.3 Biological Evaluation

##### 4.2.3.1 Animals

6–12 week‐old C57BL/6J and C57BL/6 albino mice were obtained from Charles River Laboratories (Germany) and kept until experimental procedures at the University Medical Center Mainz (Germany) with food and water supply ad libitum according to the “Guide for Care and Use of Laboratory Animals”. The performed experiments were approved by the local animal welfare authority (“Landesuntersuchungsamt Rheinland‐Pfalz”), and in vivo studies were conducted under the approval number G 20‐1‐123.

##### 4.2.3.2 Collection of Mouse Plasma

Blood was collected via heart puncture and transferred into Eppendorf tubes (1.5 mL, Sarstedt, Germany) with 1 µL Heparin (Panpharma GmbH, Germany). After centrifugation (2,000 g, 10 min, 4°C) supernatant representing plasma was taken.

##### 4.2.3.3 Isolation and Generation of Bone Marrow Derived‐Dendritic Cells (BMDCs)

Bone marrow‐derived dendritic cells were generated according to previously published procedures [36]. Briefly, bone marrow was obtained from the femur and tibia of mice by flushing out using a 20 mL syringe and a 26 G needle and test medium (IMDM containing 5% FCS, 2 mM L‐glutamine, 100 U mL^−1^ penicillin, 100 µg mL^−1^ streptomycin, 50 µM β‐mercaptoethanol). Medium and supplements were purchased from Sigma‐Aldrich (United States) and Thermo Fisher Scientific (United States). FBS was purchased from PAN‐Biotech (Germany). Single cell suspensions were generated by grinding through a 40 µm cell strainer (Greiner Bio‐One, Germany). Erythrocytes were lysed by resuspension in 1 mL Gey's Red Cell Lysis buffer (155 mM NH_4_Cl, KHCO_3_, EDTA 100 µM, pH 7.4) for 1 min at 4°C. BM cells were washed with test medium and subsequently seeded in petri dishes (2 × 10^6^/dish for GM‐CSF BMDCs and 10 × 10^6^/dish for CD103^+^ BMDCs) in 10 mL IMDM‐based test medium or RPMI‐based CD103^+^ BMDC medium (RPMI containing 10% FCS, 2 mM L‐glutamine, 100 U mL^−1^ penicillin, 100 µg mL^−1^ streptomycin, 50 µM β‐mercaptoethanol).

GM‐CSF BMDCs were generated by adding 10 ng mL^−1^ GM‐CSF (BMDC medium), and the medium was replenished on days 3 and 6 of culture. GM‐CSF BMDCs were used for subsequent uptake assays following 7 to 8 days of culture. CD103^+^ BMDCs were generated by supplementing the medium with 40 ng mL^−1^ GM‐CSF and 200 ng mL^−1^ Flt3‐L, and the medium was replenished on days 5, 9, and 12. CD103^+^ BMDCs were used for uptake assays following 13 days of culture.

##### 4.2.3.4 Isolation of Non‐Parenchymal Liver Cells (NPCs)

The non‐parenchymal liver cells (NPCs) were isolated from livers using the Liver Dissociation Kit (Miltenyi Biotech, Germany). Subsequently, NPCs were further purified by centrifugation for 15 min at 4°C and 30 g in order to separate the parenchymal cell fraction (hepatocytes) from the NPCs (remaining in the supernatant). Further purification of NPCs was achieved by density gradient centrifugation using a 30% Histodenz (Sigma–Aldrich, United States) solution as described previously [37].

##### 4.2.3.5 Isolation of Splenocytes

Splenocytes were isolated as previously described [38]. Spleens were mechanically disrupted into single cells by grinding through a 40 µm cell strainer. Erythrocytes were lysed using Gey's Red Cell Lysis buffer as described above and subsequently washed using test medium.

##### 4.2.3.6 In Vitro Cell Uptake Experiments

CD103^+^ BMDCs (10^5^ per well in CD103^+^ BMDC medium plus cytokines), GM‐CSF BMDCs (4x10^4^ in BMDC medium plus GM‐CSF) or NPCs (4.5x10^5^ per well in CD103^+^ BMDC medium w/o cytokines) were seeded in 96‐well flat‐bottom plates (all BMDCs) or 24‐well plates (NPCs). Liposomes were added at a final concentration of 1 µg mL^−1,^ and cells were incubated overnight at 37°C and 7.5% CO_2_. Finally, cells were harvested following incubation on ice (20 min) and stained for flow cytometry as described below.

##### 4.2.3.7 In Vitro Receptor Blocking Experiments

CD103^+^ BMDCs were seeded in 96‐well plates as described above. Antibodies against CD11c (clone N418) or against CLEC9A (clone 7H11) were added to the “blocking” well at a final concentration of 7.5 µg mL^−1^ and incubated for 30 min at 37°C and 7.5% CO_2_. Subsequently, liposomes were added to respective wells at a final concentration of 1 µg mL^−1^ and incubated for 3 h at 37°C and 7.5% CO_2_. Finally, cells were harvested following incubation on ice (20 min) and stained for flow cytometry as described below.

##### 4.2.3.8 In Vivo Biodistribution Experiments

Liposomes (0.5 mg mL^−1^ in 200 µL PBS) were intravenously injected into the tail veins of C57BL/6 albino mice (sample Lipo‐IgG was briefly centrifuged before injection). 3 h and 24 h following injection, whole animals were imaged via small animal fluorescence imaging (IVIS SpectrumCT, Perkin Elmer) using the following filter settings: excitation: 745 nm, emission: 800 nm. Subsequently, organs (heart, lung, liver, spleen, kidney, and inguinal lymph nodes) were dissected and imaged using the IVIS SpectrumCT. Single cell suspensions of spleens and livers (NPCs) were prepared as described above and stained for flow cytometry as described below.

##### 4.2.3.9 Flow Cytometry

Harvested cells were washed with PBS, and dead cells were stained using Live/Dead Aqua (1:1000 in PBS; Thermo Fisher Scientific) for 20 min at 4°C. Subsequently, cells were washed twice with PBS, and Fc receptors were blocked with anti‐CD16/CD32 (clone 2.4G2) for 15 min at 4°C followed by staining with fluorophore‐conjugated antibodies for 30 min at 4°C as follows. GM‐CSF BMDCs and CD103^+^ BMDCs: anti‐CD11c (clone N418). NPCs: anti‐CD11c (clone N418), anti‐F4/80 (clone BM8), anti‐CD45 (clone 30‐F11), anti‐CD172a (clone P84), anti‐Siglec‐H (clone 551), anti‐CD31 (clone 390). Splenocytes: anti‐CD11c (clone N418), anti‐CD11b (clone M1/70), anti‐CD172a (clone P84), anti‐CD8α (clone 53‐6.7), anti‐I‐A/I‐E (MHCII, clone M5/114.15.2), anti‐Siglec‐H (clone 551), anti‐CD19 (clone 6D5), anti‐CD3ε (clone 145‐2C11), anti‐CD14 (clone Sa14‐2), anti‐NK1.1 (clone PK136), anti‐Ly6G (clone 1A8). Flow cytometric analyses were performed with the Attune NxT (Thermo Fisher Scientific) and analyzed with FlowJo software v10.10.0. Gating strategies are displayed in Figures S12–S16.

##### 4.2.3.10 Statistical Analysis

Flow cytometry data were analyzed by one‐way ANOVA followed by Dunnett's multiple comparison post‐hoc test (α = 0.05), using Brown–Forsythe and Welch correction to account for unequal variances. Asterisks denote significance: ^*^ *p* < 0.05, ^**^ *p* < 0.01, ^***^ *p* < 0.001, ^****^ *p* < 0.0001, ns = not significant. All measurements were performed in triplicate and are presented as mean ± standard deviation. Analyses were performed in GraphPad Prism.

## Conflicts of Interest

The authors declare no conflicts of interest.

## Supporting information




**Supporting File**: advs74080‐sup‐0001‐SuppMat.docx.

## Data Availability

The data that support the findings of this study are available from the corresponding author upon reasonable request.
