# Supplementary material for: cDC1 Subtype‐Specific In Vivo Targeting of Liposomes
Source: Adv Sci (Weinh). 2026 Jan 28;13(19):e15402. doi: 10.1002/advs.202515402 (PMC13045298; doi:10.1002/advs.202515402)
Supplement: Supplementary file 1 — Supporting File: advs74080‐sup‐0001‐SuppMat.docx. [file ADVS-13-e15402-s001.docx]

**Supporting Information**

**cDC1 sub-type-specific in vivo targeting of liposomes**

*Maximilian Schaaf^1^, Michael Fichter^1,2^, Lin Jian^1^, Felicia Schön^2,1^, Carina Jung^1^, Paul Schneider^2^, Kai Speth^1^, Ana Mateos-Maroto^1^, Volker Mailänder^2,1^, Kaloian Koynov^1^, Svenja Morsbach^1*^, and Katharina Landfester^1*^*


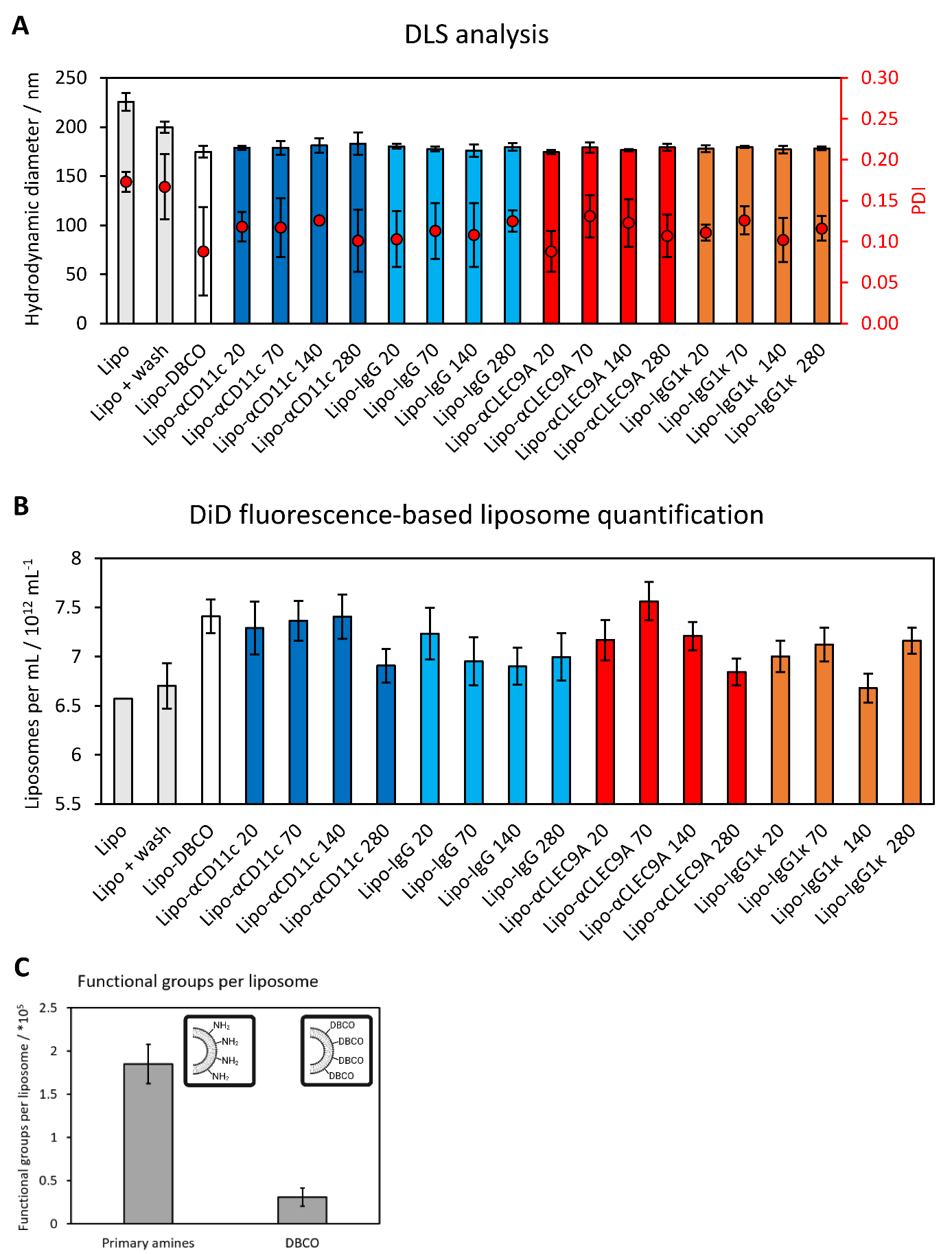


**Figure S1:** Liposome functionalization with antibodies and physicochemical characterization (batch for in vitro experiments). “Lipo” refers to unfunctionalized liposomes, “Lipo + wash” refers to unfunctionalized liposomes that were subjected to the same washing steps as performed during functionalization, “Lipo-DBCO” refers to linker-functionalized liposomes. (A) DLS analysis of liposome samples before and after antibody conjugation. (B) Liposome quantification based on DiD fluorescence signal, relative to initial Lipo sample with known concentration. (C) Functional groups per liposome as detected via fluorescamine assay and anthracene azide assay. (1.8 ± 0.2)*10^5^ NH_2_ groups per liposome before linker conjugation, (0.3 ± 0.1)*10^5^ DBCO groups per liposome after linker conjugation. Values are the mean of three replicates ± standard deviation. Created in BioRender. Schaaf, M. (2025) https://BioRender.com/vr1j9xi


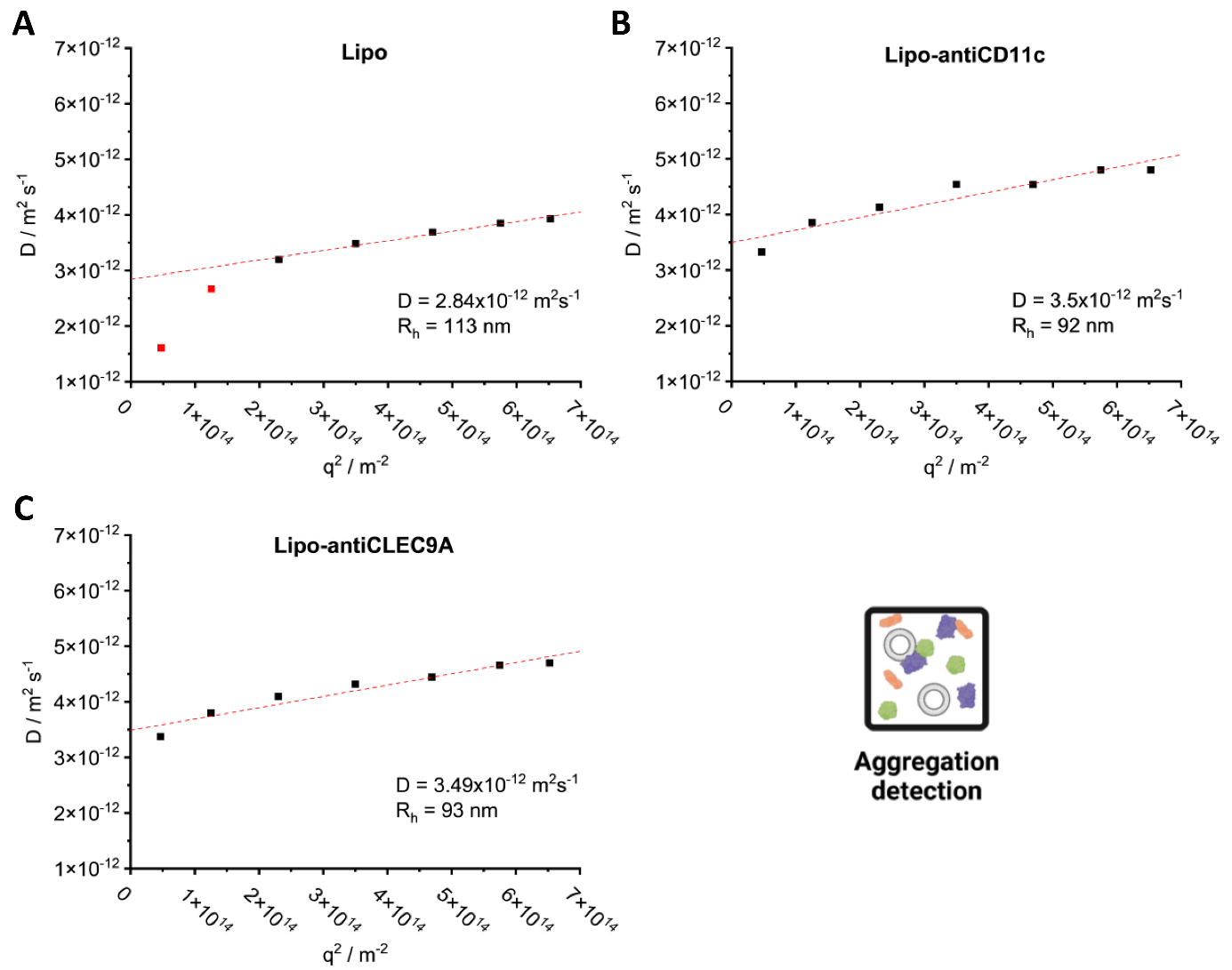


**Figure S2:** Liposome aggregation detection studies in mouse plasma at 37 °C via multi-angle DLS (batch for in vivo experiments). Graphs show the extrapolated diffusion coefficients *D* depending on scattering vector *q*^2^ for liposomes detected in plasma as detected by the fit using only the sum of individual components (liposomes + plasma). Values marked in red were not considered for the linear extrapolation. No additional aggregate formation was observed. (A) Lipo. (B) Lipo-αCD11c. (C) Lipo-αCLEC9A. Created in BioRender. Schaaf, M. (2025) https://BioRender.com/vr1j9xi


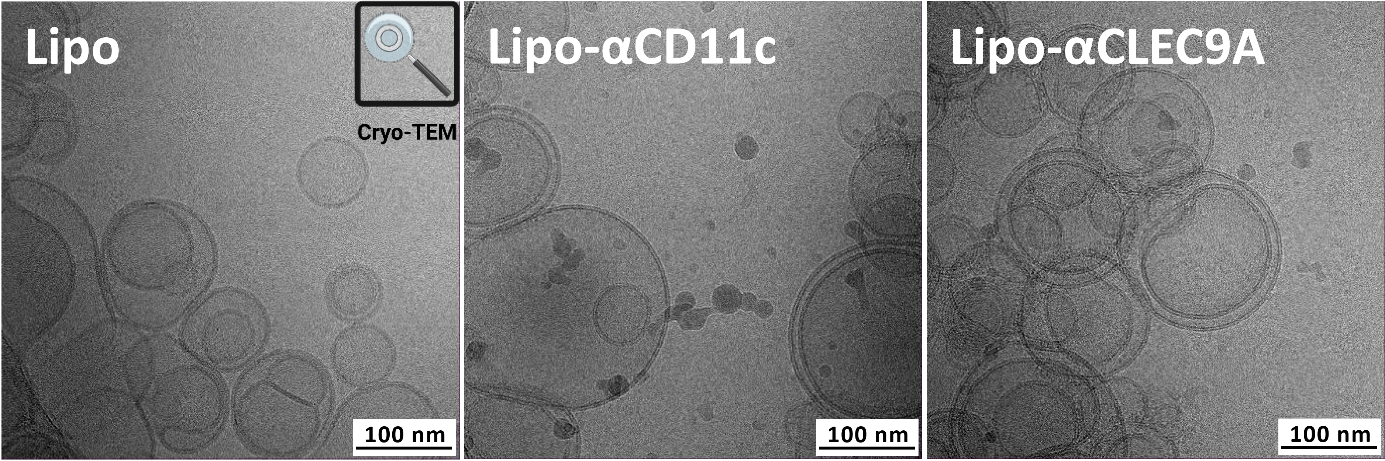


**Figure S3:** Exemplary *cryo*-TEM micrographs of liposome constructs Lipo, Lipo-αCD11c, and Lipo-αCLEC9A. Created in BioRender. Schaaf, M. (2025) https://BioRender.com/vr1j9xi


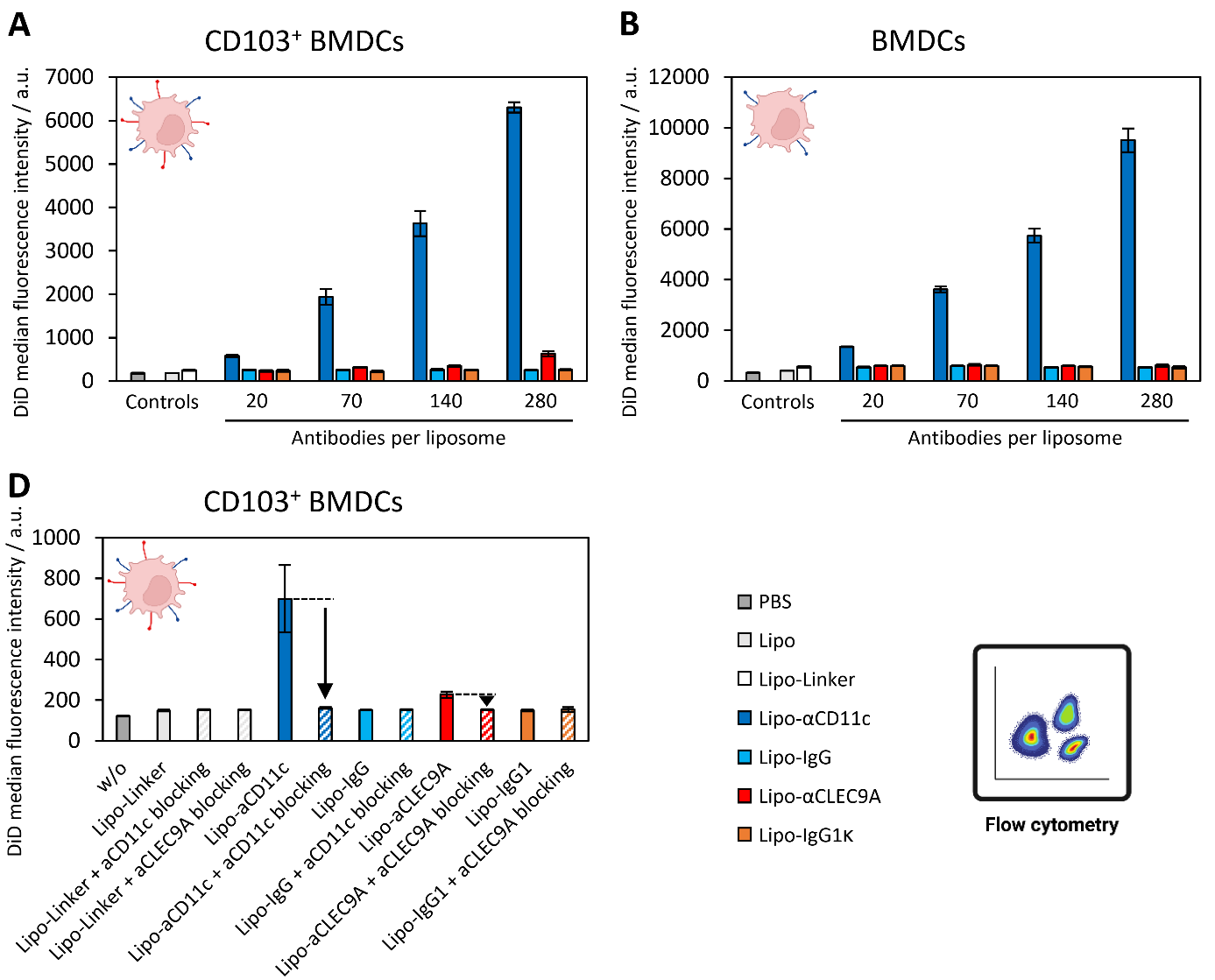


**Figure S4:** In vitro liposome cell uptake in regular or CD103^+^ BMDCs (batch for in vitro experiments). Median fluorescence intensity. (A) Liposome cell uptake in CD103^+^ BMDCs (CD11c^+^, CLEC9A^+^). (B) Liposome cell uptake in regular BMDCs (CD11c^+^, CLEC9A^-^). (C) Liposome cell uptake after cell receptor blocking in CD103^+^ BMDCs. Values are the mean of three biological replicates ± standard deviation. Created in BioRender. Schaaf, M. (2025) https://BioRender.com/vr1j9xi


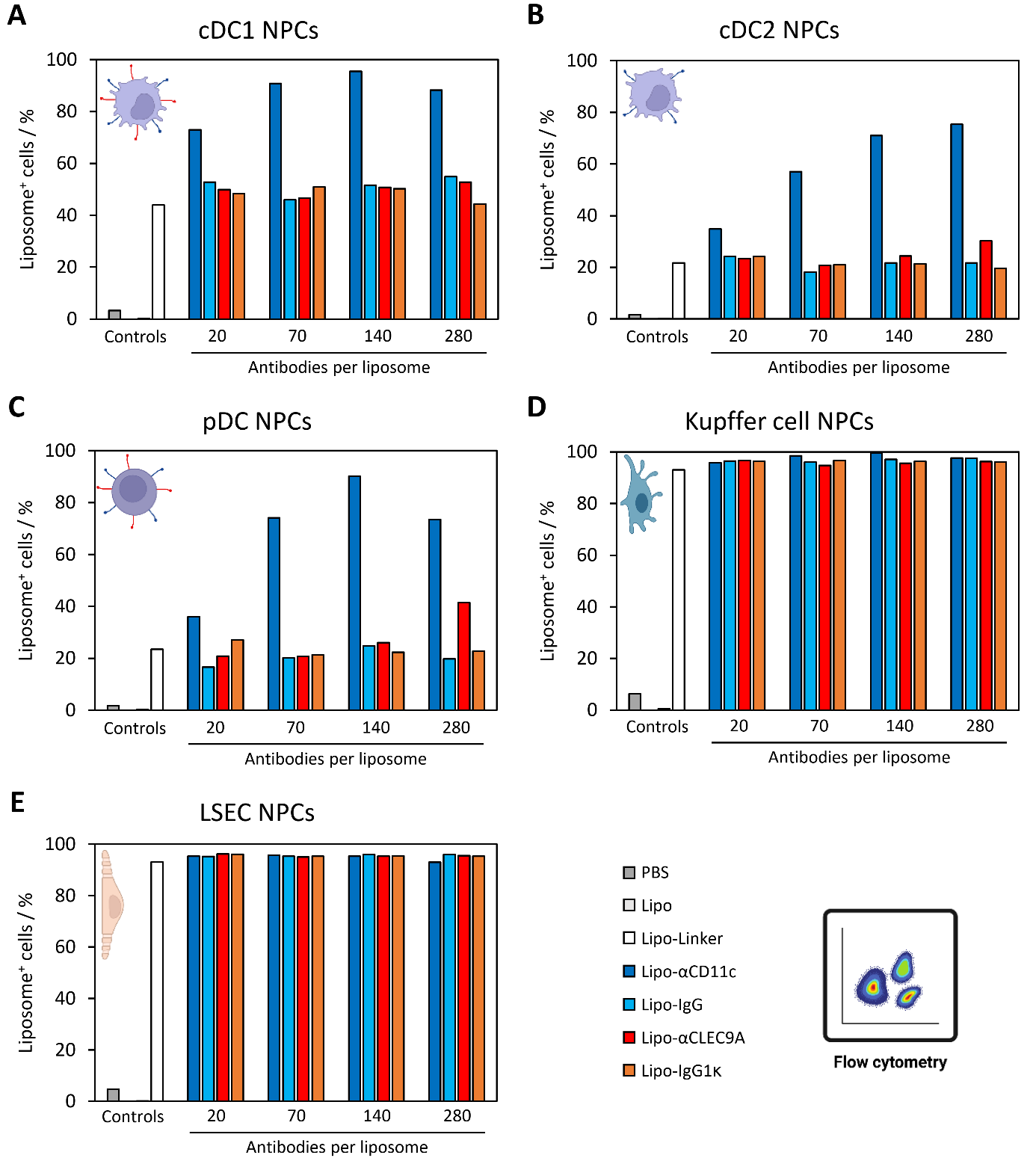


**Figure S5:** Ex vivo liposome cell uptake in liver NPCs (n=1, due to limited sample amounts). Fraction of liposome-positive cells. (A) Liposome uptake in cDC1 sub-type dendritic cells. (B) Liposome uptake in cDC2 sub-type dendritic cells. (C) Liposome uptake in pDC sub-type dendritic cells. (D) Liposome uptake in Kupffer cells. (E) Liposome uptake in LSECs. Created in BioRender. Schaaf, M. (2025) https://BioRender.com/vr1j9xi


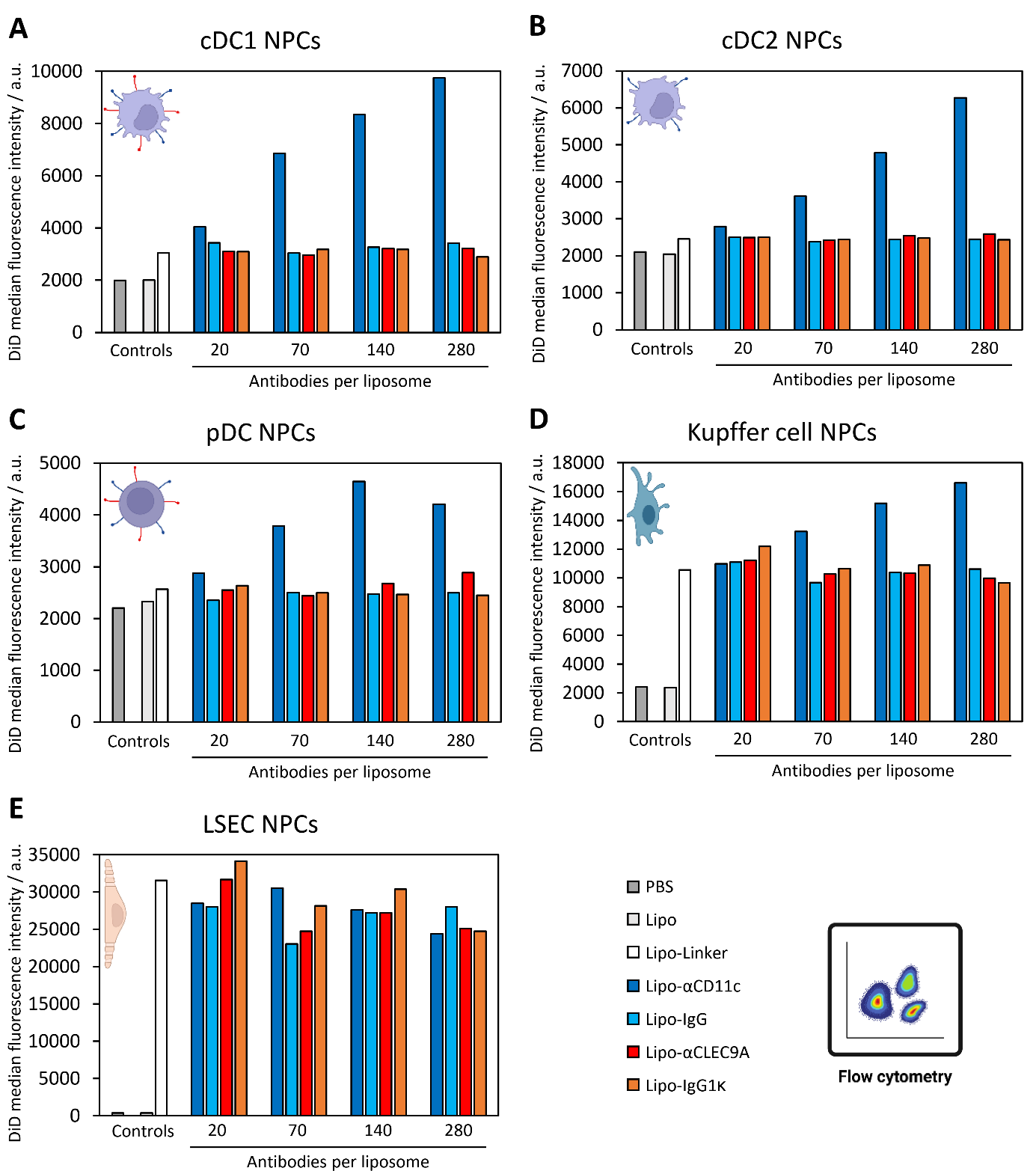


**Figure S6:** Ex vivo liposome cell uptake in liver NPCs (n=1, batch for in vitro experiments). Median fluorescence intensity. (A) Liposome uptake in cDC1 sub-type dendritic cells. (B) Liposome uptake in cDC2 sub-type dendritic cells. (C) Liposome uptake in pDC sub-type dendritic cells. (D) Liposome uptake in Kupffer cells. (E) Liposome uptake in LSECs. Created in BioRender. Schaaf, M. (2025) https://BioRender.com/vr1j9xi


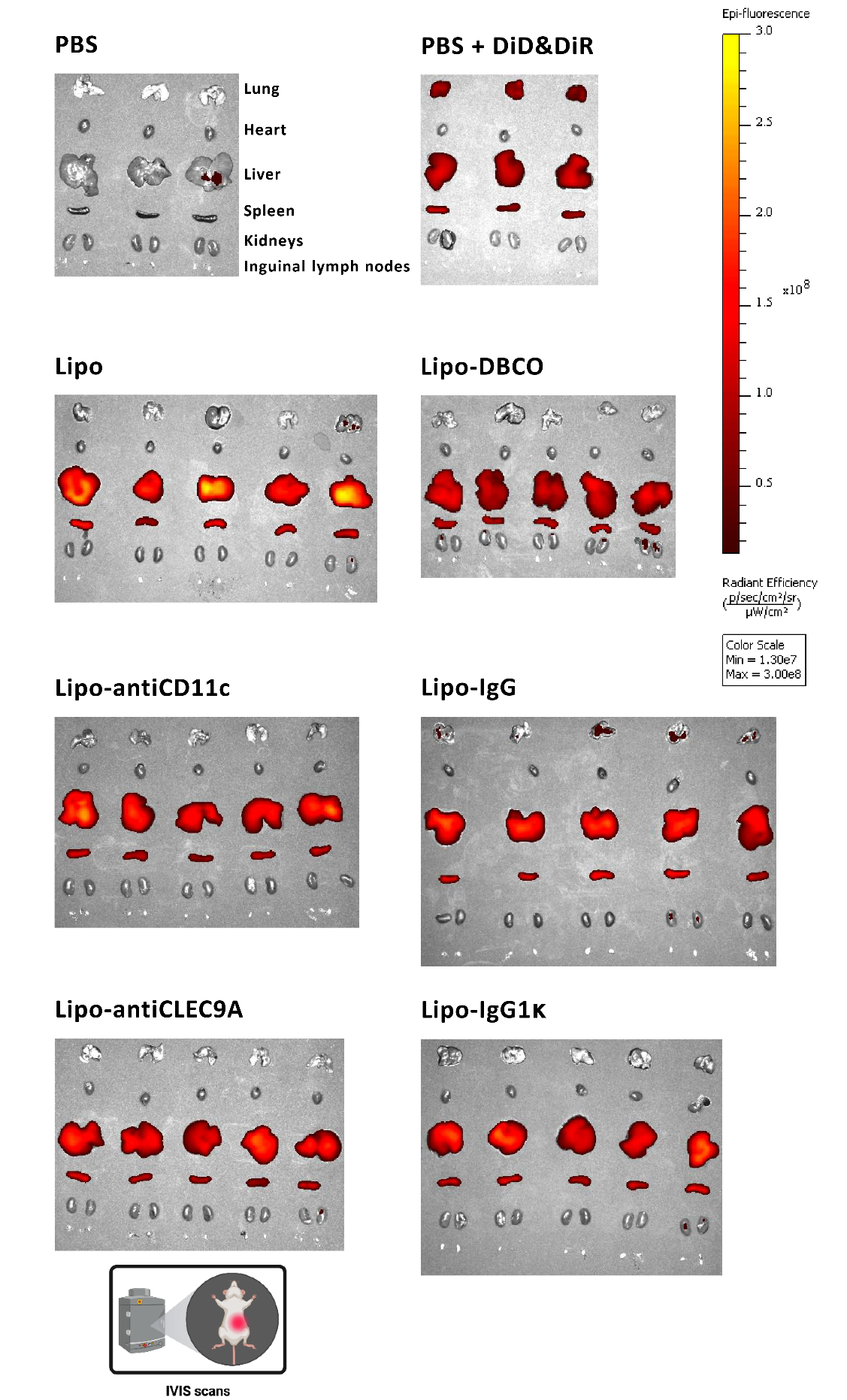


**Figure S7:** In vivo liposome biodistribution in animals injected with different liposome constructs (n=3 controls, n=5 Lipo-Abs, batch for in vitro experiments). IVIS scan images. Created in BioRender. Schaaf, M. (2025) https://BioRender.com/vr1j9xi


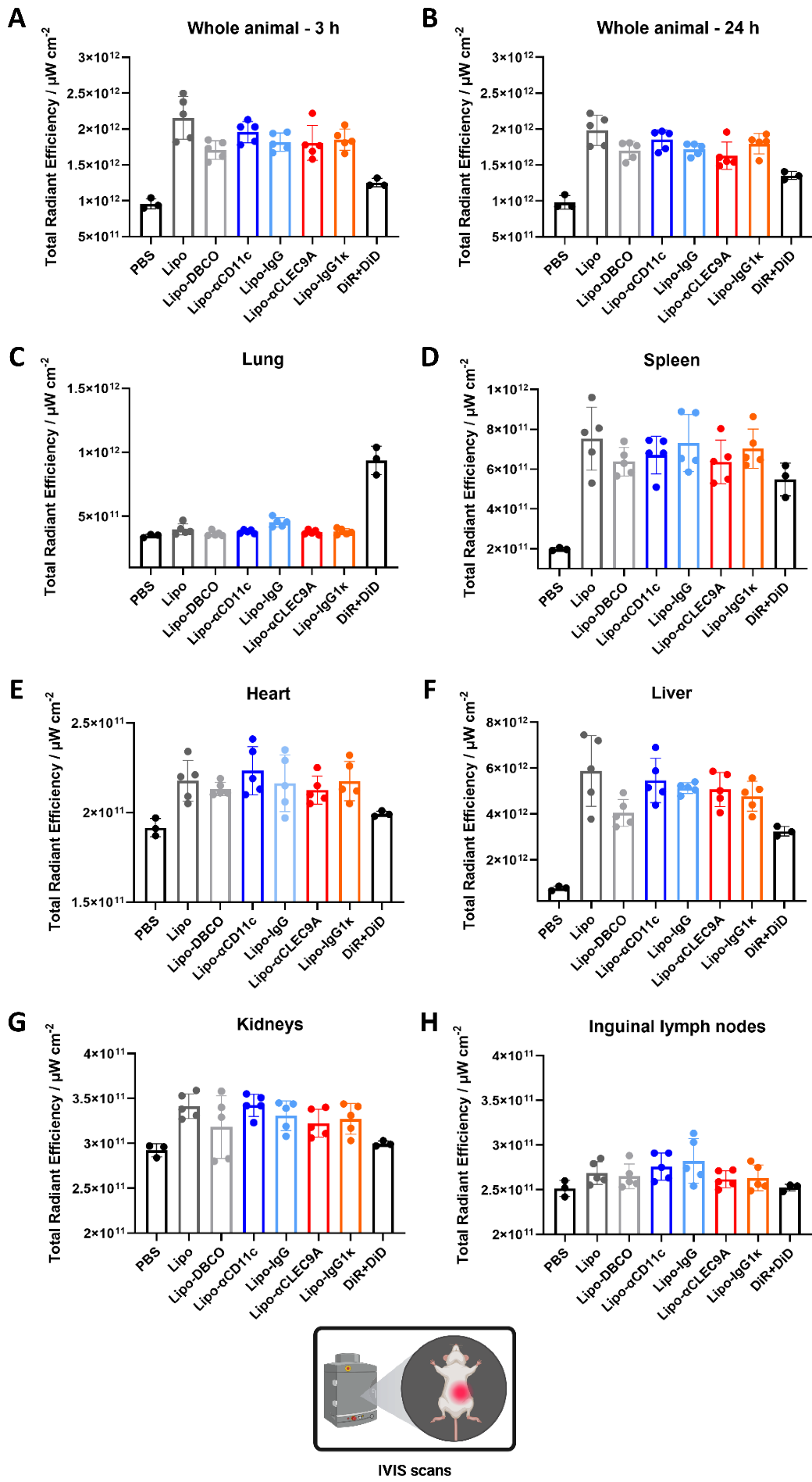


**Figure S8:** In vivo liposome biodistribution in animals injected with different liposome constructs (n=3 controls, n=5 Lipo-Abs, batch for in vitro experiments). IVIS scan of total radiant efficiency of organs. (A) Whole animal after 3 h. (B) Whole animal after 24 h. (C) Lung after 24 h. (D) Spleen after 24 h. (E) Heart after 24 h. (F) Liver after 24 h. (G) Kidneys after 24 h. (H) Inguinal lymph nodes after 24 h. Values are the mean of five biological replicates (three for PBS and DiD+DiR controls) ± standard deviation. Created in BioRender. Schaaf, M. (2025) https://BioRender.com/vr1j9xi

**
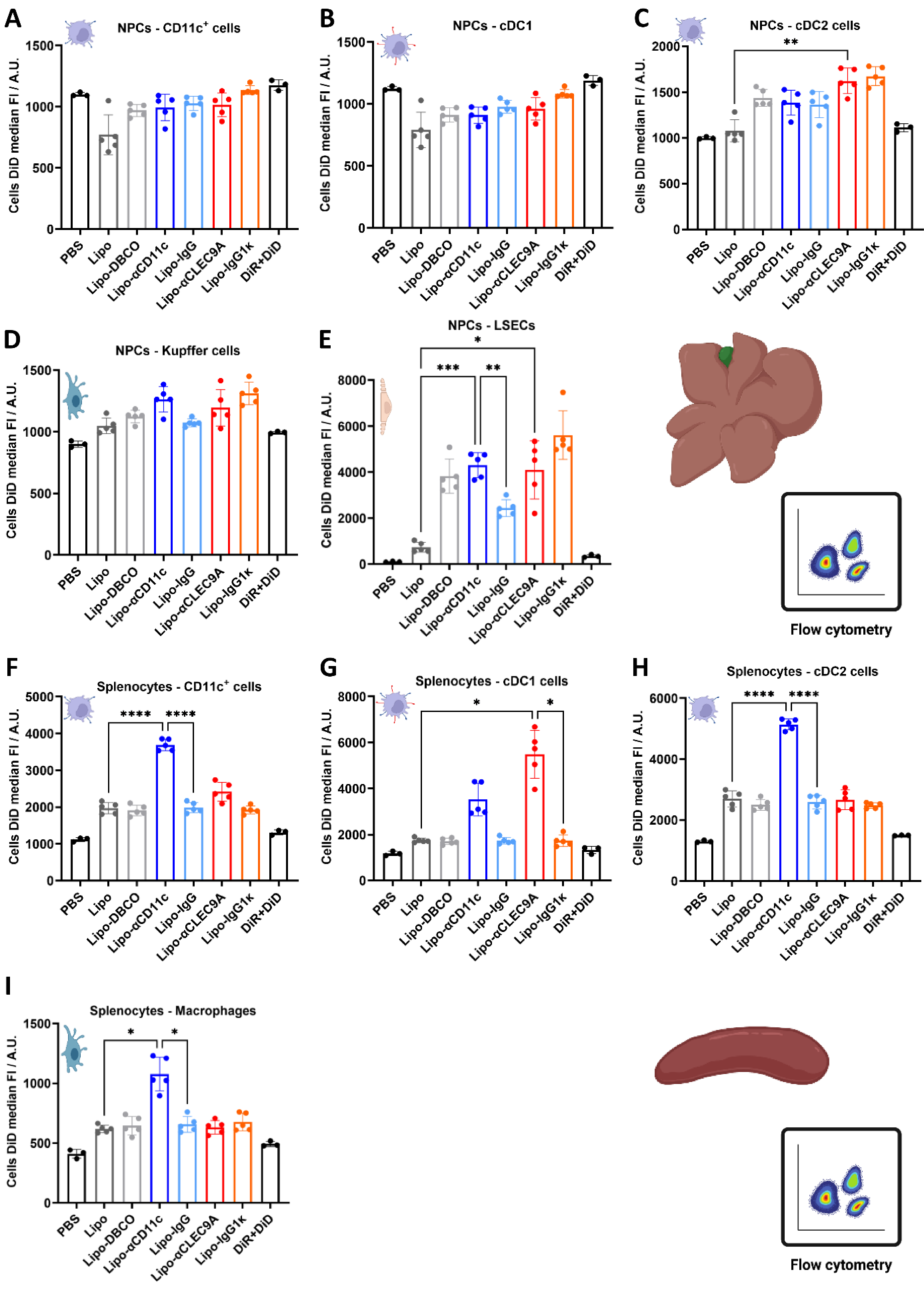
**

**Figure S9:** In vivo liposome cell uptake in liver and spleen. Median fluorescence intensity. (A) CD11c^+^ NPCs. (B) cDC1 NPCs. (C) cDC2 NPCs. (D) Kupffer cell NPCs. (E) LSEC NPCs. (F) CD11c^+^ splenocytes. (G) cDC1 splenocytes. (H) cDC2 splenocytes. (I) Macrophage splenocytes. Values are the mean of five biological replicates (three for PBS and DiD+DiR controls) ± standard deviation. Significance levels were determined through Brown-Forsythe and Welch ANOVA tests, asterisks indicate the following *p-values*: *: *p* < 0.05, **: *p* < 0.01, ***: *p* < 0.001, ****: *p* < 0.0001. Created in BioRender. Schaaf, M. (2025) https://BioRender.com/vr1j9xi


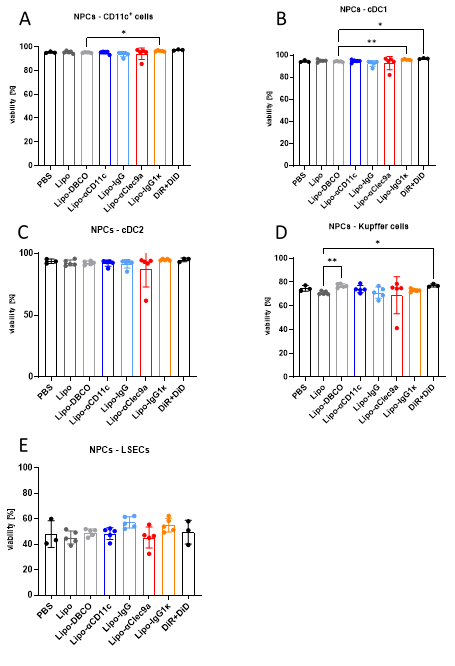


**Figure S10:** In vivo liver cell viability. (A) CD11c^+^ NPCs. (B) cDC1 NPCs. (C) cDC2 NPCs. (D) Kupffer cell NPCs. (E) LSEC NPCs. Values are the mean of five biological replicates (three for PBS and DiD+DiR controls) ± standard deviation. Significance levels were determined through Brown-Forsythe and Welch ANOVA tests, asterisks indicate the following *p-values*: *: *p* < 0.05, **: *p* < 0.01, ***: *p* < 0.001, ****: *p* < 0.0001.


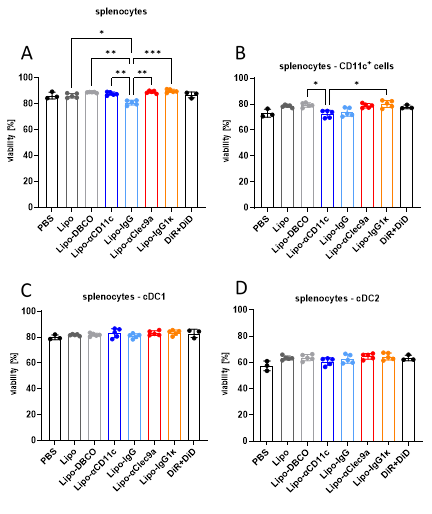


**Figure S11:** In vivo spleen cell viability. (A) All splenocytes. (B) CD11c^+^ splenocytes. (C) cDC1 splenocytes. (D) cDC2 splenocytes. Values are the mean of five biological replicates (three for PBS and DiD+DiR controls) ± standard deviation. Significance levels were determined through Brown-Forsythe and Welch ANOVA tests, asterisks indicate the following *p-values*: *: *p* < 0.05, **: *p* < 0.01, ***: *p* < 0.001, ****: *p* < 0.0001.


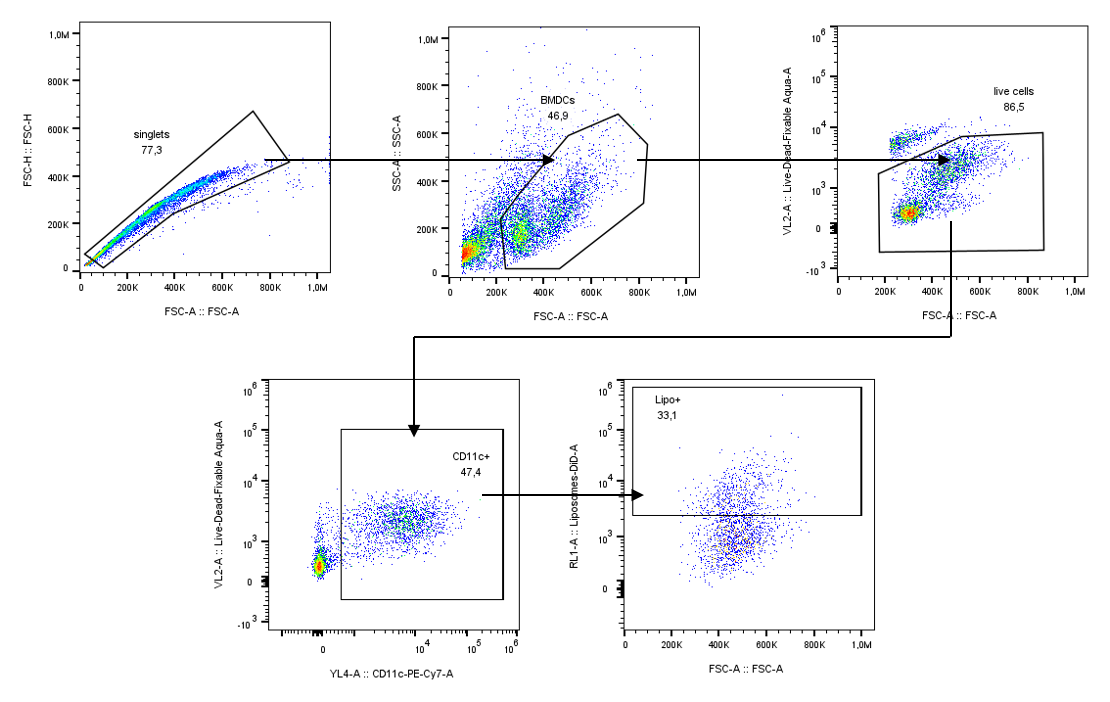


**Figure S12:** Flow cytometry gating strategy for the analysis of in vitro GM-CSF BMDCs.


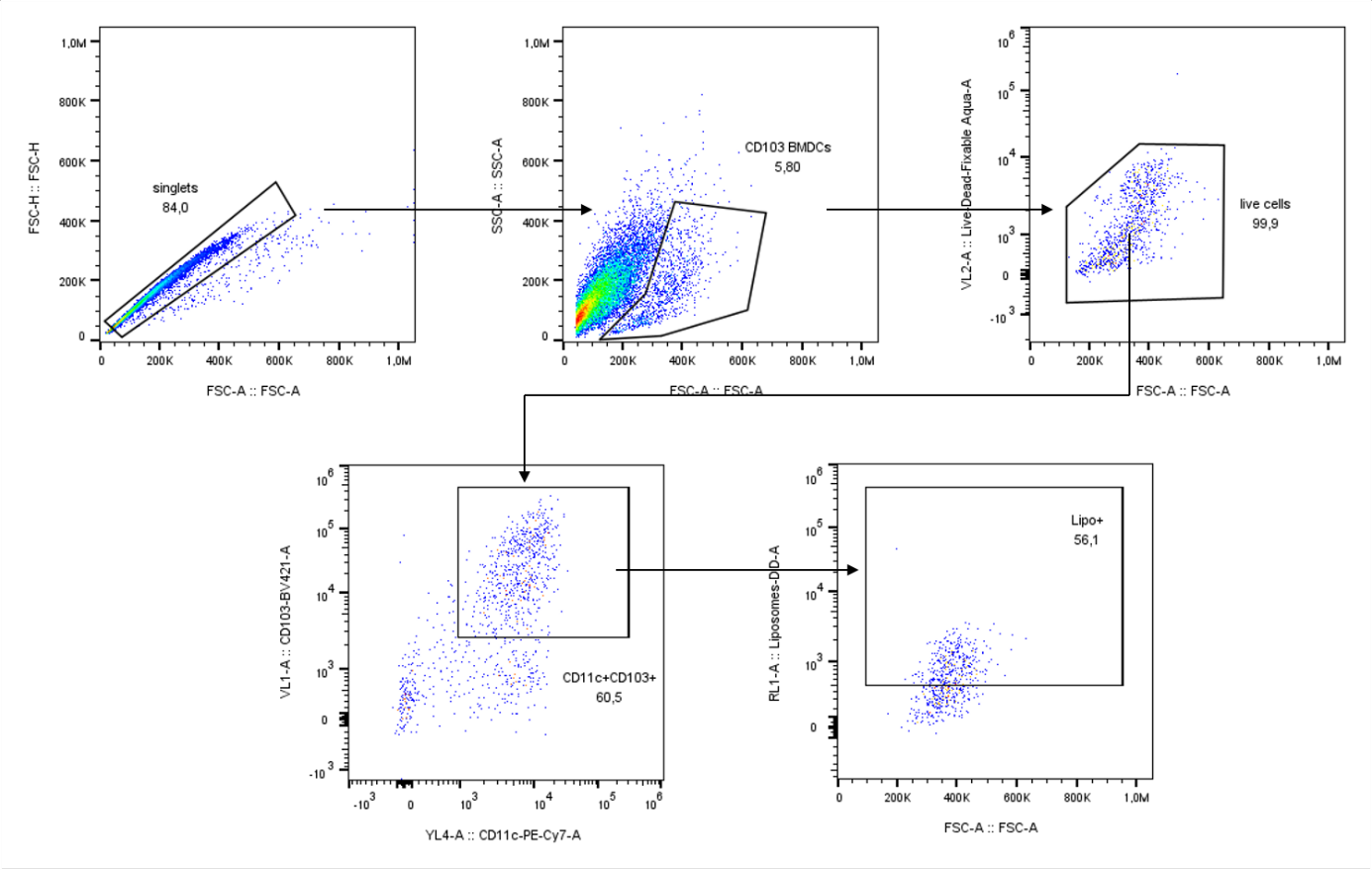


**Figure S13:** Flow cytometry gating strategy for the analysis of in vitro CD103^+^ BMDCs.


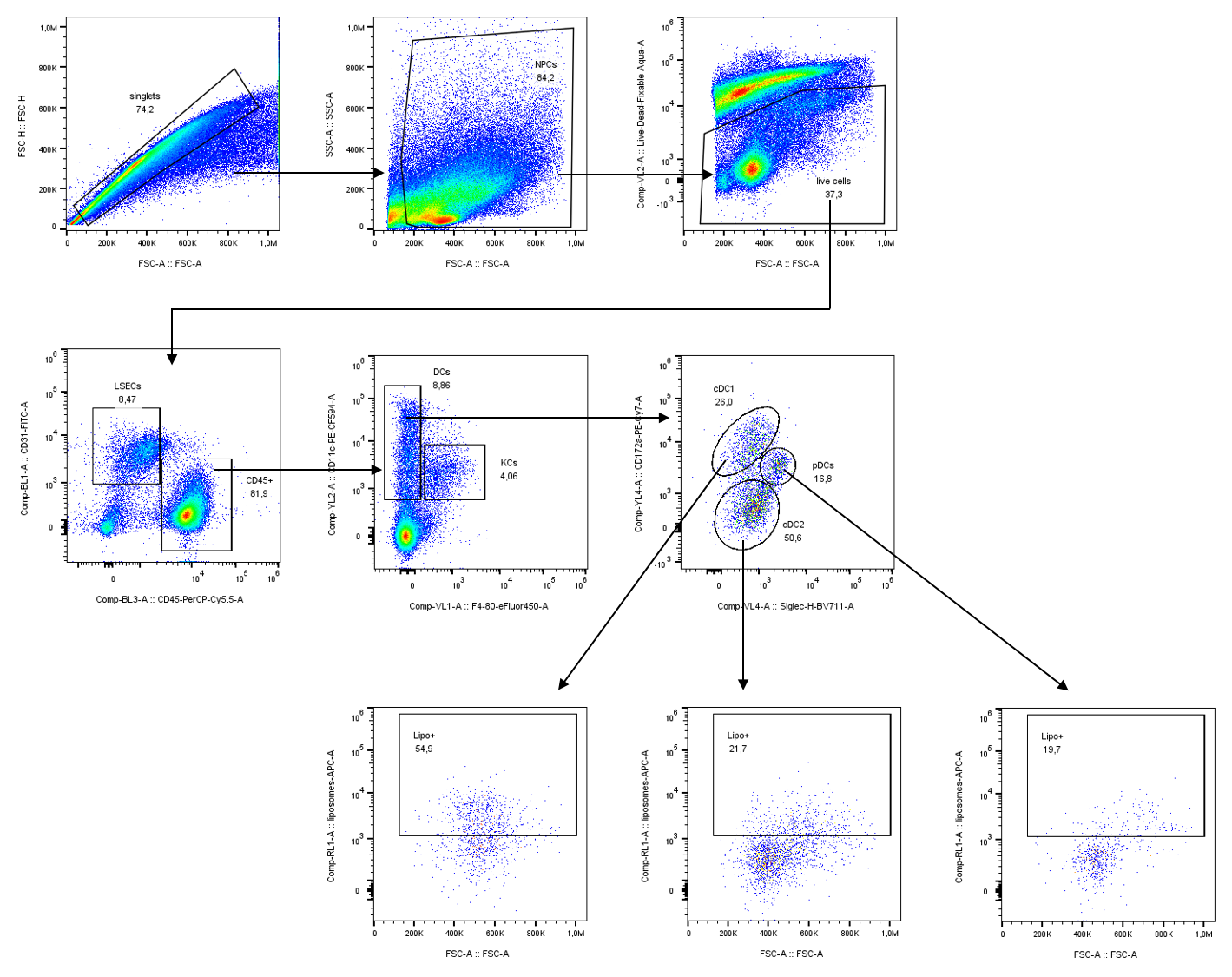


**Figure S14:** Flow cytometry gating strategy for the analysis of in vitro liver NPCs.


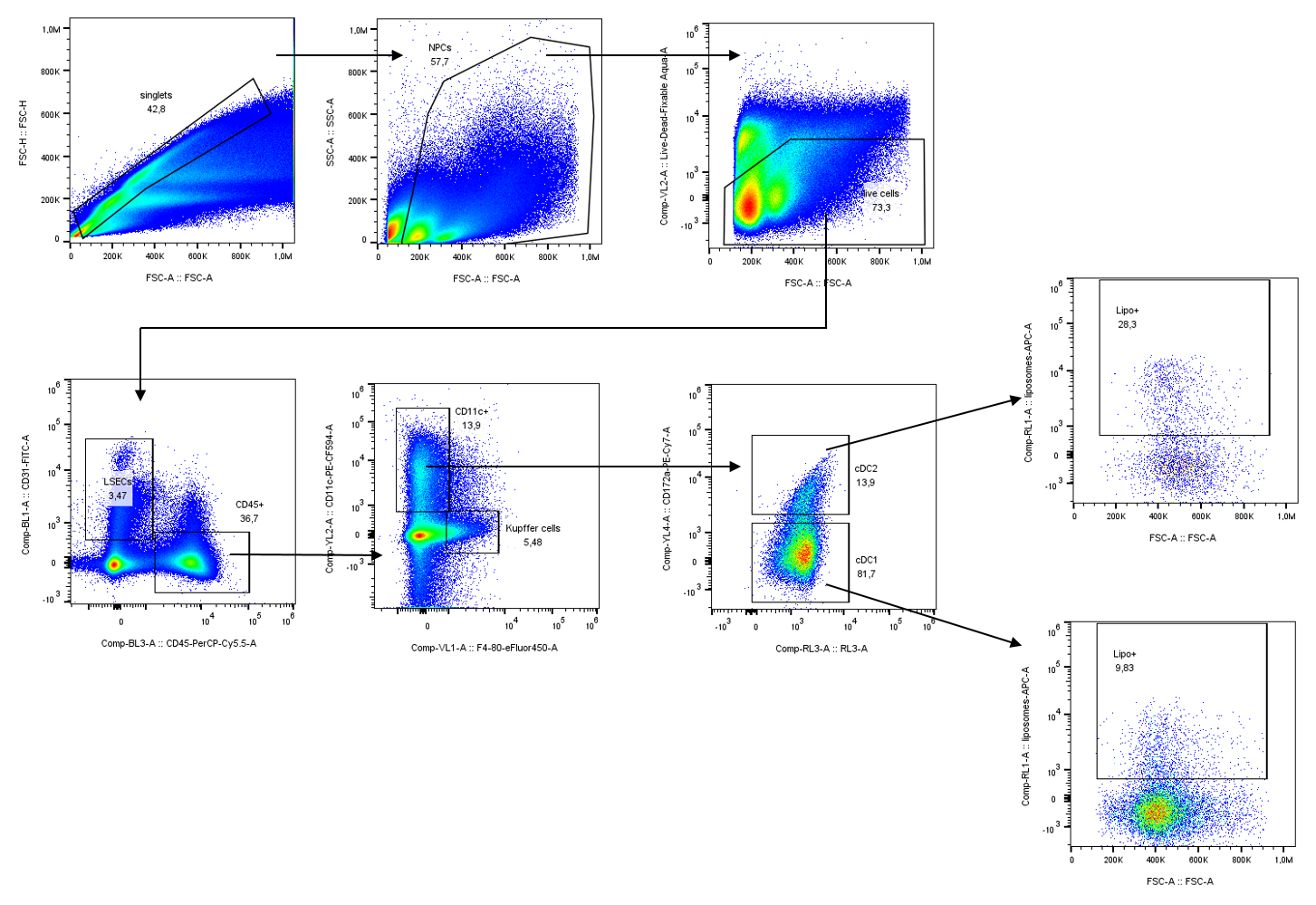


**Figure S15:** Flow cytometry gating strategy for the analysis of in vivo liver NPCs.


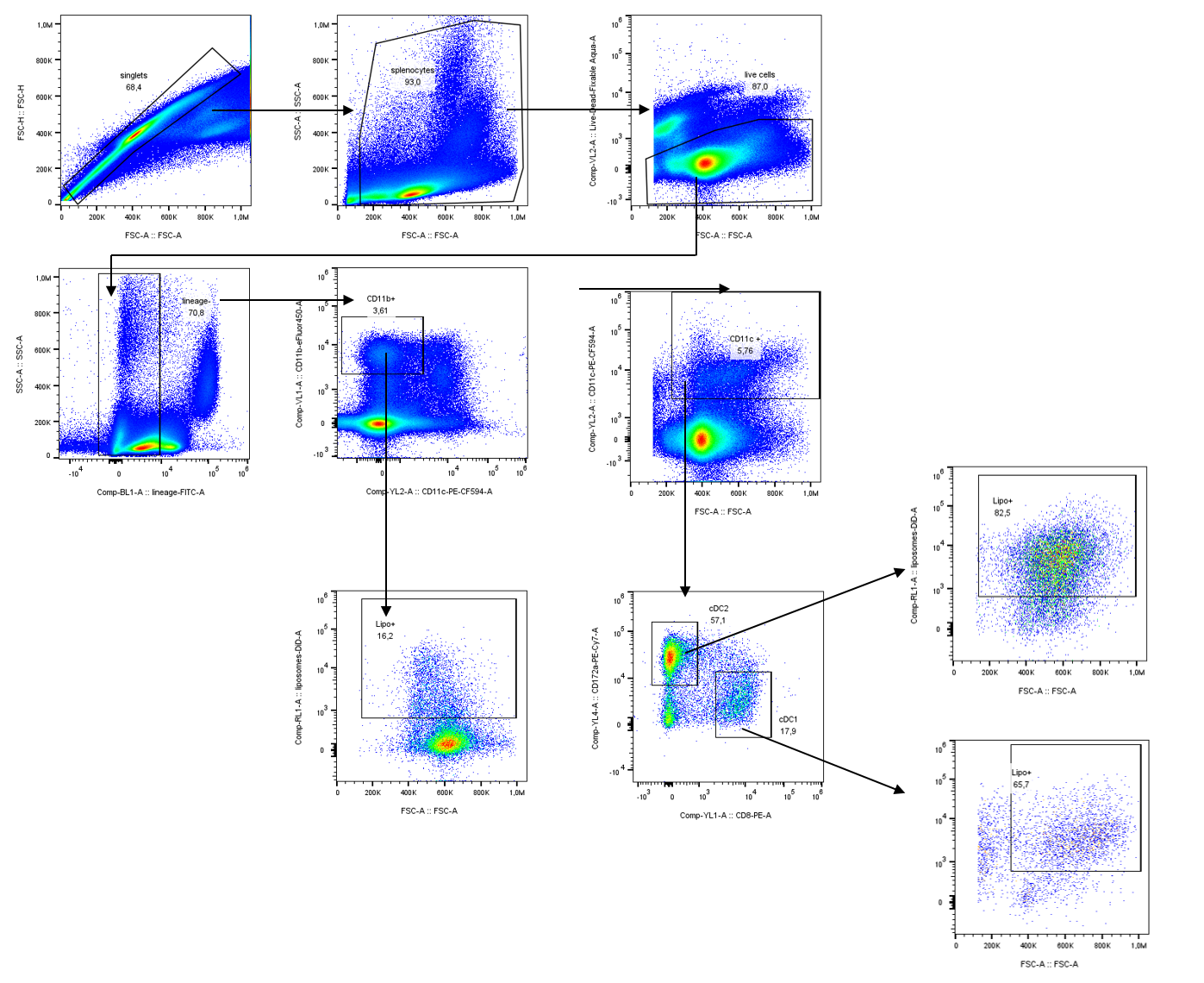


**Figure S16:** Flow cytometry gating strategy for the analysis of in vivo splenocytes.
